# A novel function of the key nitrogen-fixation activator NifA in beta-rhizobia: Repression of bacterial auxin synthesis during symbiosis

**DOI:** 10.3389/fpls.2022.991548

**Published:** 2022-09-28

**Authors:** Paula Bellés-Sancho, Yilei Liu, Benjamin Heiniger, Elia von Salis, Leo Eberl, Christian H. Ahrens, Nicola Zamboni, Aurélien Bailly, Gabriella Pessi

**Affiliations:** ^1^Department of Plant and Microbial Biology, University of Zurich, Zurich, Switzerland; ^2^Agroscope, Molecular Ecology and Swiss Institute of Bioinformatics, Zurich, Switzerland; ^3^ETH Zürich, Institute of Molecular Systems Biology, Zurich, Switzerland

**Keywords:** root-nodule, legume, indole-acetamide, nitrogenase, infection

## Abstract

Rhizobia fix nitrogen within root nodules of host plants where nitrogenase expression is strictly controlled by its key regulator NifA. We recently discovered that in nodules infected by the beta-rhizobial strain *Paraburkholderia phymatum* STM815, NifA controls expression of two bacterial auxin synthesis genes. Both the *iaaM* and *iaaH* transcripts, as well as the metabolites indole-acetamide (IAM) and indole-3-acetic acid (IAA) showed increased abundance in nodules occupied by a *nifA* mutant compared to wild-type nodules. Here, we document the structural changes that a *P. phymatum nifA* mutant induces in common bean (*Phaseolus vulgaris*) nodules, eventually leading to hypernodulation. To investigate the role of the *P. phymatum iaaMH* genes during symbiosis, we monitored their expression in presence and absence of NifA over different stages of the symbiosis. The *iaaMH* genes were found to be under negative control of NifA in all symbiotic stages. While a *P. phymatum iaaMH* mutant produced the same number of nodules and nitrogenase activity as the wild-type strain, the *nifA* mutant produced more nodules than the wild-type that clustered into regularly-patterned root zones. Mutation of the *iaaMH* genes in a *nifA* mutant background reduced the presence of these nodule clusters on the root. We further show that the *P. phymatum iaaMH* genes are located in a region of the symbiotic plasmid with a significantly lower GC content and exhibit high similarity to two genes of the IAM pathway often used by bacterial phytopathogens to deploy IAA as a virulence factor. Overall, our data suggest that the increased abundance of rhizobial auxin in the non-fixing *nifA* mutant strain enables greater root infection rates and a role for bacterial auxin production in the control of early stage symbiotic interactions.

## Introduction

Rhizobia are nitrogen (N_2_)-fixing symbiotic bacteria that trigger the formation of root organs in leguminous plants, called nodules, where biological nitrogen fixation (BNF) takes place (Poole and Allaway, 2000; Masson-Boivin et al., 2009; Oldroyd et al., 2011). The establishment of this symbiotic interaction relies on a sophisticated molecular dialog between bacterial and plant partners (Lee and Hirsch, 2006; Ferguson et al., 2010; Oldroyd et al., 2011; Via et al., 2016). Plants first exude flavonoids in the rhizosphere, which chemoattract rhizobia to the roots (Lee and Hirsch, 2006; Cooper, 2007; Ferguson et al., 2010; Via et al., 2016). In response to flavonoids, bacteria induce the expression of the *nod* (nodulation) genes responsible for the biosynthesis of lipochitooligosaccharides known as nodulation factors (NFs). The perception of NFs leads to root hair deformation and curling that entrap the bacteria in an infection pocket (Gage and Margolin, 2000; Gage, 2004; Ferguson et al., 2010; Oldroyd et al., 2011). In parallel, NFs activate a signaling cascade in the plant cells that leads via a calcium spiking response to massive cell division in the root cortex. Then, multiplying bacteria penetrate the infection threads (ITs) formed toward the nodule primordia, where rhizobia are released into plant cells and differentiated into N_2_-fixing bacteroids (Gage, 2004; Lee and Hirsch, 2006; Oldroyd et al., 2011; Poole et al., 2018). The expression of the rhizobial *nif* (nitrogen fixation) genes is nodule-specific and is activated by the alternative RNA polymerase sigma factor σ^54^ (or RpoN) together with its enhancer binding protein NifA (Hauser et al., 2007; Lardi et al., 2018). The impact of the loss of NifA function on nodulation, in addition to the absence of nitrogenase activity (Fix^–^), has been described in several legume symbioses. Despite the different nodule types, the Fix^–^ nodules triggered by *nifA* mutant symbionts were often reported impaired in leghemoglobin production, to structurally decay, and/or eventually turn necrotic. For instance, the roots of *Lotus japonicus* colonized with the *Mesorhizobium loti* MAFF303099 *nifA2* mutant display minute white nodules (Nukui et al., 2006) while *Medicago truncatula* plants inoculated with the *Sinorhizobium meliloti* 1021 *nifA* mutant develop Fix^–^ mature nodules but, in contrast to wild-type symbionts, the bacteria prematurely die shortly after bacteroid elongation (Berrabah et al., 2015). A *Bradyrhizobium diazoefficiens* strain USDA110 *nifA* mutant showed an increased number of nodules all over the soybean root system and the induced nodules were necrotic (Fischer et al., 1986; Parniske et al., 1991). During symbiosis with *Aeschynomene americana*, the Fix^–^ *Bradyrhizobium* sp. DOA9 *nifAp nifAc* double mutant induced smaller nodules without impacting nodulation frequency (Wongdee et al., 2018). These typical Fix^–^ phenotypes led to suboptimal host plant development, hence indicating that NifA function is essential for symbiotic nitrogen fixation and nodule maintenance (Berrabah et al., 2015).

Phytohormones, especially auxin and cytokinin are also involved in coordinating the nodulation process (Ferguson et al., 2010; Oldroyd et al., 2011; Liu et al., 2018; Velandia et al., 2022). Auxins were first discovered as plant hormones playing crucial roles in plant growth, development, embryogenesis and tropism (Zhao, 2010). Several studies have shown that auxin transport and localization are important for nodule organogenesis during rhizobia-legume symbiosis since auxin influences nodule initiation, differentiation and vascular bundle formation (Mathesius, 2008; Kohlen et al., 2018; Shrestha et al., 2020; Torres et al., 2021). Interestingly, certain rhizobia are also capable of synthetizing auxins, mostly indole-3-acetic acid (IAA), which can directly interfere with plant auxin homeostasis (Mathesius, 2008; Spaepen and Vanderleyden, 2011) and functions as a signaling molecule (Spaepen et al., 2007; Spaepen and Vanderleyden, 2011). The bacterial pathways for IAA synthesis either depend on or are independent of tryptophan (TRP) as precursor (Duca et al., 2014; Zhang et al., 2019). Although the TRP-independent pathway is not well studied yet, five pathways have been categorized as TRP-dependent: the indole-3-acetamide (IAM), the indole-3-pyruvic acid (IPA), the tryptamine, the indole-3-acetonitrile and the tryptophan side-chain oxidase pathways (Spaepen et al., 2007; Zhang et al., 2019). In microorganisms, the two most common and better studied pathways are the IAM and IPA pathways (Supplementary Figure 1; Spaepen et al., 2007; Morffy and Strader, 2020). In the IAM pathway that is mainly present and used by phytopathogenic bacteria, a monooxygenase (encoded by *iaaM*) converts TRP into IAM, which is then hydrolyzed to IAA and ammonia by the IAM hydrolase (encoded by *iaaH*). The IPA pathway converts TRP into IAA in three steps. First an aminotransferase converts TRP to IPA that is then decarboxylated to indole-3-acetaldehyde (IAAld) by the indole-3-pyruvate decarboxylase IpdC. IPA is finally oxidized to IAA by the IAAld dehydrogenase (Spaepen and Vanderleyden, 2011; Zhang et al., 2019; Morffy and Strader, 2020). Although this pathway can be found in some plant pathogens, it has been mostly associated with beneficial bacteria (Spaepen et al., 2007; Spaepen and Vanderleyden, 2011; Patten et al., 2013). In rhizobia-legume symbiosis, bacteria contribute to the production of IAA in nodules (Spaepen et al., 2007; Spaepen and Vanderleyden, 2011). Studies on *Rhizobium leguminosarum* bv. *viciae* LPR1105 genetically engineered to express *iaaM* from *Pseudomonas savastanoi* and *tms2* from *Agrobacterium tumefaciens*, suggested that IAA plays a role in the maintenance of a functional root nodule in *Vicia hirsute*, since nodules of this genetic variant exhibited a more efficient nitrogenase activity, a bigger size and a delayed senescence (Camerini et al., 2008).

*Paraburkholderia phymatum* STM815 is one of the main model organisms to study beta-rhizobia-legume symbiosis (Moulin et al., 2001) and stands out for its ability to establish N_2_-fixing symbiosis with a wide range of legumes, including the economically important *Phaseolus vulgaris* (common bean) (Elliott et al., 2006; Gyaneshwar et al., 2011; Mishra et al., 2012; Moulin et al., 2014; Lemaire et al., 2016; Lardi et al., 2017a). We previously showed that *P. phymatum* NifA is essential for nitrogenase activity during symbiosis and that a *P. phymatum nifA* mutant induced an increased number of *P. vulgaris* nodules compared to plants infected with the wild-type strain (Lardi et al., 2017b). Furthermore, a combined metabolomics and dual RNA-sequencing analysis of common bean nodules induced by a *P. phymatum nifA* mutant, revealed that NifA down-regulates the expression of *P. phymatum*’s auxin genes *iaaM* and *iaaH* thus leading to IAM and IAA accumulation. In contrast to the above-mentioned studies on legume symbioses with different rhizobial *nifA* mutants, the loss of NifA in *P. phymatum* did not trigger an immune response against the mutant bacteria (Bellés-Sancho et al., 2021a).

Here, we further investigate the effect in the absence of NifA on nodule architecture as well as the role of NifA in controlling the expression of *iaaMH* during different stages of symbiosis, from the plant-rhizobia recognition step until the development of a mature root nodule. Our data showed that nodules induced by the *P. phymatum nifA* mutant developed normally despite their clustered organization. We show that NifA represses the expression of the *iaaMH* genes in all stages of symbiosis. Furthermore, by constructing a mutant strain lacking the *iaaMH* operon, we demonstrated that *P. phymatum* produces IAA via the IAM pathway inside *P. vulgaris* root nodules. While this pathway does not seem to be essential for a functional symbiosis, the up-regulation of *iaaMH* in a strain lacking NifA was responsible for the increased number of nodule clusters. Finally, orthologs of the *P. phymatum iaaMH* genes were found in many strains of important phytopathogens such as *Pseudomonas syringae* and *Agrobacterium tumefaciens*, suggesting a related role in plant infection.

## Materials and methods

### Bacterial strains, media, and cultivation

Bacterial strains, primers and plasmids used in this work are listed in Supplementary Table 1. *Escherichia coli* and *P. phymatum* strains were routinely cultivated in Luria-Bertani (LB) (Miller, 1972) media and its modified version without salt (LB-NaCl), respectively (Liu et al., 2020). *Escherichia coli* strains were grown aerobically at 37°C and 220 rpm, while *P. phymatum* strains were incubated aerobically at 28°C and 180 rpm. When required, the growth medium was supplemented with the following antibiotics at the indicated concentrations: chloramphenicol (Cm, 20 μg/mL for *E. coli* and 80 μg/mL for *P. phymatum*), kanamycin (Km, 25 μg/mL for *E. coli* and 50 μg/mL for *P. phymatum*) or trimethoprim (Tm, 50 μg/mL for *E. coli* and 100 μg/mL for *P. phymatum*). For the *gfp*-expression analysis with germinated seeds, bacteria were grown on (A) BG medium [(A) B-minimal media supplemented with 0.3 mM NH_4_Cl and 10 mM glucose as carbon source] (Liu et al., 2020).

### Construction of *Paraburkholderia phymatum* GFP reporter strains and mutant strains

To construct the promotor fusion, the promotor region of Bphy_7769 was amplified by PCR with Bphy_7769p_*Eco*RI_F and Bphy_7769p_*Sal*I_R primers, using as a template *P. phymatum* STM815 genomic DNA (gDNA). The 207 bp-long product was digested with the restriction enzymes *Eco*RI and *Sal*I and cloned into the multiple cloning sites in front of the *gfp* gene in the vector pPROBE-NT using the same restriction sites as the insert. After transformation in *E. coli* Top10, the cloned sequence was verified by sequencing at Microsynth (Balgach, St. Gallen, Switzerland). The constructed plasmid was transferred into *P. phymatum* wild-type and a *nifA* insertional mutant (Lardi et al., 2017b) by triparental mating. The deletion mutant of the *iaaMH* genes was constructed by cloning an antibiotic resistance gene in between two external flanking sequences of the genes to be deleted. The primers Bphy7769_up_F_*Eco*RI and Bphy7769_up_R_*Nde*I were used to amplify the Bphy_7769 upstream fragment of 529 bp and the primers Bphy7768_dn_F_XhoI and Bphy7768_dn_R_*Eco*RI for the 525 bp-length to get the Bphy_7768 downstream fragment. A trimethoprim cassette *dhfr* followed by a transcription terminator was digested with *Nde*I from the p34E-TpTer plasmid (Shastri et al., 2017) and cloned in between the upstream and downstream fragments. The resulting 1,945 bp long DNA fragment was then inserted into the suicide pSHAFT2 vector, resulting in the pSHAFT2:Δ*iaaMH* plasmid, which was verified by sequencing. The pSHAFT2:Δ*iaaMH* plasmid was transferred into *P. phymatum* wild-type by triparental mating. Clones sensitive to Cm and resistant to Tm were selected and purified. The genomic integration of the construct was verified by PCR using Bphy7770_veri_F and Bphy7767_veri_R primers. The auxin deletion mutant was called Δ*iaaMH*. To construct the *nifA*-Δ*iaaMH* double mutant, the pSHAFT-nifA plasmid (Lardi et al., 2017b) was conjugated in *P. phymatum* Δ*iaaMH* and clones resistant to Cm and Tm were selected. To generate the complemented strain of the Δ*iaaMH* mutant and *nifA*-Δ*iaaMH* double mutant, both *iaaM* and *iaaH* genes with their native promoter were amplified by PCR using the primers Bphy7769_comp_F_*Xba*I and Bphy7768_comp_R_*Xba*I. The 3,246 bp-length product was then digested with *Xba*I and cloned in forward and reverse direction into the multicloning site of the vector pBBR1MCS2 with respect to the direction of *lacZ*α gene. These two *iaaMH* complemented constructs differ on their promoters: while the expression of *iaaMH* cloned in forward direction is driven by the promotor of *lacZ*α (called pBBR1MCS2:*iaaMH_*_*p*_*lacZ*), the transcription of *iaaMH* genes cloned in reverse direction relies on their native promotor (pBBR1MCS2:*iaaMH*). The constructed Δ*iaaMH* or *nifA*-Δ*iaaMH* complemented strains were called Δ*iaaMH* + pBBR-*iaaMH* and *nifA*-Δ*iaaMH* + pBBR-*iaaMH* when *iaaMH* was expressed by their natural promoter or Δ*iaaMH*/*nifA*-Δ*iaaMH* + pBBR-*iaaMH*(_*p*_*lacZ*) when they were under the control of the strong promoter. The correct sequence was verified by sequencing using the LacZ_R and Bphy7768_seq_R primers.

### Seedling preparation, plant infection, and growth condition

*Phaseolus vulgaris*, cv. Negro jamapa (common bean) seeds were surface-sterilized with absolute ethanol for 5 mins, 35% solution of H_2_O_2_ for another 5 mins and subsequently washed 10 times with sterile deionized water (Talbi et al., 2010). Seeds were placed on 0.8% agarose plates and incubated 48 h at 28°C in the dark for germination. Sprouted seeds were then transferred into yogurt-jars containing sterile vermiculite (VTT-Group, Muttenz, Switzerland) and 160 mL of nitrogen-free Jensen media (Hahn and Hennecke, 1984). Bacterial strains were grown in liquid LB-NaCl overnight as previously described. Then, cultivated cells were collected, washed twice with (A)B-minimal medium (Liu et al., 2020) and adjusted to a final OD_600_ of 0.025 (10^7^ cells per mL) (Lardi et al., 2017b). One mL was inoculated directly on each germinated seedling. Plants were grown in the green-house with a day/night temperature of 25/22°C with 16 h of light in a constant humidity of 60%. Plants were watered twice a week with sterile deionized water and harvested after 21 days of incubation.

### Characterization of symbiotic properties *in planta*

To assess the symbiotic properties, nodules of 21 days old plants inoculated with *P. phymatum* strains were harvested. The number of nodules, dry weight per nodule and nitrogenase activity were determined as previously described (Lardi et al., 2017a,b). To obtain the nodule occupancy, at least one nodule per inoculum was surface sterilized as previously described (Lardi et al., 2017a). Nodules were crushed and bacteroids were isolated by plating on LB-NaCl and counting of the colony forming units (CFU). For the quantification of the nodule-clustering phenotype, each root system (*n* = 15 plants) was completely washed with deionized water and individual basal roots were severed from the primary root and vertically aligned onto one polyester clear sleeve per plant. The individual root systems were scanned (300 dpi, ImageScanner III, GE Healthcare) and the following parameters were recorded from the images with ImageJ. We measured the primary and basal root lengths and numbers and recorded for every nodule of each root its dimensions (ROI area and shape descriptors) and its position on the root axis relative to the proximal extremity of the root.

### Histological preparations

To obtain precise cytological features, the cell walls of *P. vulgaris* nodules were stained with Calcofluor White M2R (Sigma-Aldrich, Buchs, St. Gallen, Switzerland). In brief, fresh nodules midpoint transversal sections were obtained under the binoculars with sterilized fine blades and immediately covered with 20 μl of a filter-sterilized 1 mg/mL Calcofluor White solution in deionized water. After 5 mins of incubation, excess of dye, cell debris and free bacteria were removed by washing thrice with sterile tap water. The sectioned nodules were directly mounted in 10% glycerol on glass slides for acquisitions. Live/Dead staining was performed using the BacLight Bacterial Viability Kit (L7012, Invitrogen, Bleiswijk, Netherlands) according to the manufacturer guidelines. The procedure was applied to bacteroids extracted from fresh nodules after disrupting the tissue in 400 μl sterile 0.9% NaCl with a disposable micropestle and 2 mins centrifugation at 1,500 rpm, or to bacteria recovered from bacteroids into selective medium. For direct assessments of bacteroids viability into host cells, live/dead straining procedure was performed simultaneously to Calcofluor White preparations. All images were obtained from a confocal laser scanning microscope (DM5500Q; Leica, Wetzlar, Germany) fitted with a TCS SPE confocal unit (Leica, Wetzlar, Germany), an ACSAPO 40 × oil-immersion objective (NA = 1.15, Leica, Wetzlar, Germany) or an ACSAPO 10 × dry objective (NA = 0.3, Leica, Wetzlar, Germany), and laser lines set at 405, 488, and 532 nm with fixed detection windows corresponding to the maximum emission peaks of the respective fluorophores, using the LAS software (Leica, Germany). In each experimental set, the acquisition parameters were identical between samples. The obtained images were analyzed with ImageJ.^1^

### *In planta gfp*-expression analysis

To visualize the induction of *iaaMH* expression on the surface of germinated bean roots, overnight cultures of *P. phymatum gfp*-reporter strains with empty pPROBE, _*p*_*nodB*, and _*p*_*iaaMH* in the wild-type and *nifA* mutant strain were washed twice with 10 mM MgSO_4_ and inoculated in melted 0.8% agarose (A)BG medium to a final OD_600_ of 0.05. Germinated seeds were placed in the middle of the plate with the tip of the primary root submerged into the medium while the agarose medium was still liquid. After solidification, plates were incubated for 3 days at 28°C. Three independent biological replicates were prepared for this assay. The fluorescence was then quantified using a Leica M205 FCA fluorescent stereo microscope equipped with a DFC 7000 T CCD camera and the relative fluorescent signal was acquired through an ET GFP filter set (470/40 nm excitation, 525/50 nm emission). The GFP expression in bean inoculated with the *P. phymatum gfp*-reporter strains was monitored 10, 14, and 21 days after inoculation. Next, the sections were placed on a glass slide and observed under the microscope. Deeper inspection of the tissue sections was performed using the confocal microscope with additional Calcofluor White staining when required. The obtained images were analyzed using ImageJ.

### Metabolite extraction and data analysis

The metabolite abundance in *P. vulgaris* root nodules inoculated with different *P. phymatum* auxin-mutant strains was compared. As previously described, approximately 30 mg of nodules or uninfected roots were immediately frozen with liquid nitrogen and metabolites were extracted using cold methanol (Lardi et al., 2016, 2018; Bellés-Sancho et al., 2021a,b). Three independent biological inoculants were processed with one or two plants per inoculum. Next, the extracts were injected twice and analyzed by non-targeted flow injection-time-of-flight mass spectrometry on an Agilent 6550 QTOF instrument (Agilent Technologies, Santa Clara, CA, USA), using the settings previously described (Fuhrer et al., 2011). A total of 285 ions were detected with distinct *m/z* within a tolerance of 0.001 Da and were matched to expected deprotonated molecules. The abundance of the metabolites involved in IAA production [according to the KEGG database (Kanehisa and Sato, 2020)] were estimated and compared according to the ion count values for each sample. The complete metabolomics data set, including a list of detected ions, annotations and intensities, is listed in the Supplementary Table 2.

### Auxin production quantification

The estimation of the amount of auxinic compounds secreted by *P. phymatum* wild-type, *nifA* mutant, *iaaMH* mutant and the corresponding *iaaMH* complemented strains, was performed as previously described (Bellés-Sancho et al., 2021a). Briefly, overnight cultures were routinely grown in LB-NaCl and cells were collected by centrifugation (5 mins at 5,000 rpm) and washed twice with LB-NaCl. An initial cell density of OD_600_ = 0.05 was inoculated into 20 mL of LB-NaCl per strain and cultivated for 16 h at 28°C with 180 rpm shaking. Supernatants were collected by centrifugation and mixed with Salkowski’s reagent in a volume ratio of 1:2 (Gordon and Weber, 1951). After 20 mins of incubation in the dark, the mixtures’ absorbances were measured at 535 nm and compared to a standard curve of pure IAA (1003530010, Sigma-Aldrich, Buchs, St. Gallen, Switzerland). Three biological replicates were performed per strain.

### Bioinformatic analysis

To identify bacteria that encode orthologs of the *iaaMH* genes and orthologs of *nifA*, *nifH*, and *nodA* (in *P. phymatum* STM815, these genes are all located on the 595,108 bp symbiotic plasmid pBPHY02), the protein sequences of IaaM (WP_012406795.1), IaaH (WP_012406794.1), NifA (WP_012406756.1), NifH (WP_012406781.1), and NodA (WP_012406745.1) were searched together against the NCBI’s Identical Protein Groups (IPG) resource using cblaster (Gilchrist and Chooi, 2021). The maximum intergenic distance allowed between conserved hits was set to 600 kb and the presence of at least IaaM and IaaH was required, using cblaster’s default BLAST parameters (e-value threshold of 0.01, minimum protein identity of 30% and query coverage of 50%). To visualize genes between and in the vicinity of these five query genes, the intermediate genes option was enabled and synteny plots of the 150 best-scoring clusters were generated using cblasters plot_cluster mode. By default, cblaster only reports orthologs that are assigned to a cluster, meaning they cannot exceed the specified maximum intergenic distance and must occur on the same contig, a limitation for scaffold-level assemblies. Therefore, the cblaster session file was parsed using a custom Python script to extract both BLAST hits that could be assigned to a cluster as well as those that could not. Specifically, for each strain identified by cblaster, each query protein on top of IaaM and IaaH was checked for having a hit located in a cluster. If at least one hit was part of a cluster, the most similar hit to the query in the cluster was chosen. Otherwise, the hit with overall highest similarity was chosen, regardless of its genomic position. Similarity was defined as the product of sequence coverage and sequence identity, both normalized to 1 (range 0.15–1.0). Strains were grouped by species and the minimal and maximal cluster score assigned by cblaster (based on the number and similarity of genes in the cluster), as well as the minimal and maximal similarity for each gene was calculated. The resulting summary table (Supplementary Table 3) was grouped by genus and species and ordered by their respective maximum cluster scores. The table was then filtered to only contain assigned species (all “sp.” entries were removed) and some additional entries were removed (having a contamination, not being a genome assembly or failing NCBI’s taxonomy check). A phylogenetic tree based on the IaaH and IaaM orthologs in the species listed in Supplementary Table 3 was generated. A single genome was chosen to represent each species: The NCBIs representative genome was chosen if it was identified by cblaster, otherwise the best genome was selected based on NCBIs taxonomy check, completeness of the genome, and the number of contigs reported. The orthologs of both proteins were separately aligned using Clustal Ω 1.2.4 (Sievers et al., 2011) and the automatic model selection mode of RAxML 8.2.12 (Stamatakis, 2014) (PROTGAMMAAUTO) was used to determine the best protein selection model for each protein. The alignments of both genes were concatenated using BioPython 1.79 (Cock et al., 2009) and RAxML was used to create 100 bootstrap trees with partitions defined such that the best model is used for each protein (LG for IaaH and JTT for IaaM, still in PROTGAMMA mode). The resulting bootstrap trees were concatenated and bootstrap values were added to the best tree using RAxML with -f b flag. The tree was visualized using FigTree. To look at conservation on the DNA level, the nucleotide sequence from Bphy_7758 to Bphy_7769 (NC_010627.1:528492-542282) was blasted against NCBIs nt database (downloaded 05.05.2022) using blastn 2.12.0 + with the default settings from NCBIs website. The results were converted to GFF format using the NCBIXML module from BioPython and inspected in IGV (Thorvaldsdóttir et al., 2013). The GC content in the region of the predicted *P. phymatum iaa* operon (Bphy_7767-9; Burkholderia genome database^2^; NC_010627:532782-547282) was analyzed using a custom Python script as a sliding window analysis with a window size of 2 kb and a step size of 0.4 kb. A second sliding window analysis with a window size equal to the operon length of 4,500 bases was used to determine if the GC content of the predicted *iaa* operon significantly deviated from the average GC content of windows of similar size from the plasmid pBPHY02 (NC_010627). The GC content of the predicted operon was calculated separately and its location among all sorted window GC contents was determined. The results were visualized with Matplotlib 3.5.1 (Hunter, 2007; Caswell et al., 2021).

### Statistical analysis

The statistical analysis for the characterization of the *gfp* expression data, symbiotic properties, Salkowski’s indolics quantification and the metabolite’s ion count analysis of the nodules was performed with GraphPad Prism 6.0 using an ordinary one-way ANOVA with Tukey’s multiple comparison (*p*-value ≤ 0.05). 1D clustering of the pooled nodules positions was performed according to the Jenks natural breaks classification method with 10 classes and 25,000 iterations. A population of 500 randomized values ranging from 0 to 250 mm was used as negative control.

## Results

### *Paraburkholderia phymatum* lacking *NifA* (Fix^–^) does not develop abnormal nodules in common bean

**FIGURE 1:**
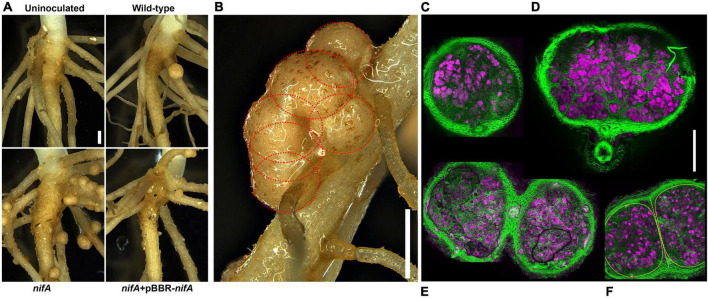
*nifA*-triggered nodules that fuse during development. **(A)** Representative root-shoot junctions of 11 days post inoculation (dpi) *Phaseolus vulgaris* inoculated with or without the indicated strains. Bar = 1 mm. **(B)** Close-up view of an unusual structure formed by *Paraburkholderia phymatum nifA*-occupied nodules. The red dotted circles delineate the boundaries of six independent but intertwining nodules. Bar = 1 mm. **(C–F)** Confocal laser scanning micrographs of nodular transversal sections. Maximum z-projections of 30 μm × 1 μm optical sections. Calcofluor white staining of plant cell walls (green) and Syto9 DNA staining of bacteroids (magenta). Single wild-type or *nifA*-triggered young (**C**, here colonized by wild-type *P. phymatum*) or mature (**D**, here colonized by *P. phymatum nifA* mutant) nodules are indistinguishable at macroscopic scale, respectively. **(E)** Two closely developing *nifA* nodules sharing cortical and vascular tissues (black lines delineate air bubbles produced in the mounting of the sample). **(F)** Fused *nifA* nodules (yellow outlines) separated by a cortical wall (dashed line). Bar = 500 μm.

The Fix^–^ *P. phymatum* STM815 *nifA* mutant triggers hypernodulation in *P. vulgaris* (Lardi et al., 2017b), a phenotype we associated with the repression of the autoregulation of nodulation (AON) system (Bellés-Sancho et al., 2021a). To evaluate the structural effects on common bean nodules induced by the *P. phymatum nifA* mutant, 11-days old bean plants inoculated with the *nifA* mutant were compared to inoculations with wild-type and *nifA* complemented symbionts. The examined bean plants infected with the *nifA* mutant harbored an increased number of nodules that often clustered in mature parts of the roots (Figure 1A) and unusual structures. This phenotype was reverted by genetic complementation of the *nifA* mutant. Closer inspection of the mature atypical structures under the microscope revealed that they were in fact composed of manifold nodules that seem fused with each other (Figure 1B). Transversal sections of single young, round nodules and larger, flatter mature nodules displayed no notable histological differences between plants infected with wild-type and *nifA*, and their overall organization remained consistent with established determinate nodule architectures (Figures 1C,D). However, the sections of clustered *nifA* nodules showed various levels of fusion of cortical, parenchymal and endodermal tissues, shared vasculature but distinct infection zones, *i.e.*, originating from discrete ITs (Figures 1E,F). We did not find any *nifA* nodule with necrotic or disorganized internal architecture. Mature wild-type, *nifA* and *nifA* complemented nodules indistinctively exhibited the coloration characteristic of leghemoglobin. The organogenesis of nodules occupied with the *nifA* mutant strain was thus not impaired and the observed cluster-like structures seem to be derived from multiple primordia initiated at very close sites.

**FIGURE 2:**
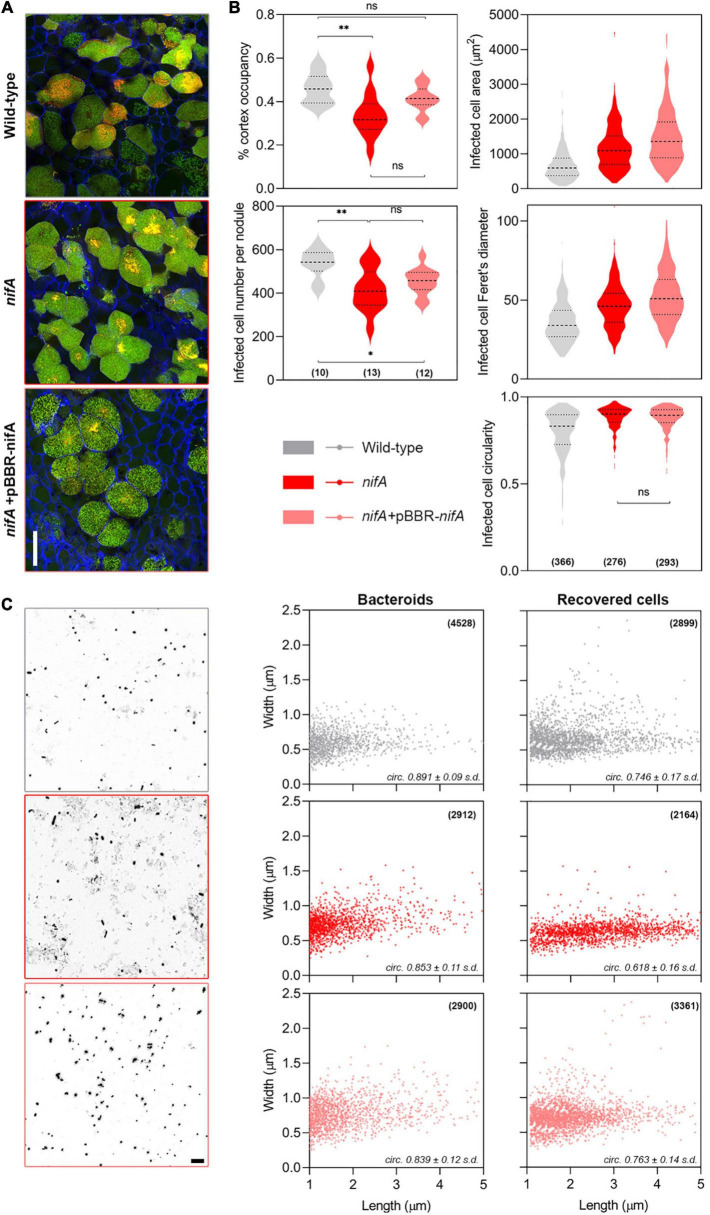
Phenotypic characterization of *Phaseolus vulgaris* nodules occupied by *Paraburkholderia phymatum* wild-type, *nifA* and *nifA*-complemented strains. **(A)** Live/Dead staining of infected cells. Confocal laser scanning micrographs of nodular transversal sections. Maximum z-projections of 10 μm × 0.5 μm optical sections. Propidium iodide (red), Syto9 (green), and Calcofluor White (blue). Note the similar distribution of red/green signals between the different samples. All samples are shown at the same magnification, bar = 50 μm. **(B)** Nodules and plant cells parameters. Numbers in brackets indicate the number of independent samples. Data represent two independent assays. Ordinary one-way ANOVA with Tukey’s *post hoc* test. The absence of annotation between samples implies *p* < 0.001. Otherwise ^**^p < 0.01, *p < 0.05, ns, not significant. **(C)** Bacterial cells parameters. Left, high-contrast bright field micrographs of bacteroids preparations obtained from crushed fresh nodules. Bar = 10 μm. Right, cytometric properties of bacteroids obtained from crushed nodules and bacteroids cultured in selective medium. Cell dimensions were extracted from CLSM micrographs with MicrobeJ. Numbers in brackets indicate sampled cells number. The calculated cell circularity is indicated in italics. *s.d.*, standard deviation of mean.

*In situ* live/dead staining of mature nodule transversal sections confirmed that wild-type, *nifA* and *nifA* complemented bacteroids occupying infected plant cells showed comparable survival rates, with a similar distribution of live/dead cells (Figure 2A and Supplementary Figure 2). However, we observed that nodules colonized by the *nifA* mutant showed a decreased number of infected cells and an overall lower occupancy of the nodular tissues. Further, plant cells infected by the *nifA* mutant appeared bigger, longer and rounder than wild-type infected cells (Figure 2B). Interestingly, these alterations were not totally mitigated in nodules occupied by the *nifA* complemented strain. As cell death of *nifA* bacteroids occurs in elongating cells (Berrabah et al., 2015), we sought to compare the morphologies of the wild-type and mutant bacteroids obtained from surface-sterilized, crushed nodules. *P. phymatum* STM815 bacteroids appeared as a mixture of rod-shaped and spherical cells, irrespective of the strain identity, with a significant number of bacteroids undergoing cell division (Figure 2C). We also recovered the bacteroids by growing them on fresh medium with appropriate antibiotic selection and subjected them to the same analysis. All strains grew as rod-shaped bacteria with similar dimensions to the corresponding bacteroids, with a slight increase in dividing cells number for the *nifA* mutant. It is noteworthy to mention that live/dead staining of these cells did not show significant differences between strains (data not shown). Our data suggest that *P. vulgaris* nodules occupied by *nifA* do not drastically differ from wild-type colonized nodules. The only noticeable difference we could associate with NifA loss were disturbances in infected plant cell size and frequency.

### *Paraburkholderia phymatum iaaMH* are expressed in presence of germinated seeds and repressed by NifA

**FIGURE 3:**
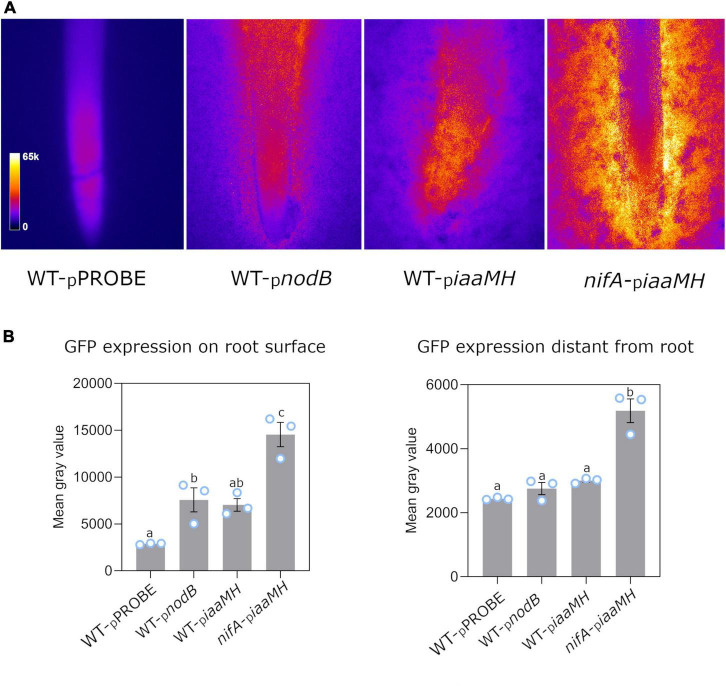
NifA-dependent *iaaMH-gfp* expression on germinated *Phaseolus vulgaris* roots in *Paraburkholderia phymatum* STM815 wild-type (WT-_*p*_*iaamH*) and in a *nifA* mutant (*nifA*-_p_*iaaMH*). A *gfp* reporter construct was used. *P. phymatum* wild-type containing the empty pPROBE (WT-_*p*_PROBE) and the *nodB* promoter sequence (WT-_*p*_*nodB*) were used as a negative and a positive control, respectively. **(A)** Representative stereomicrographs of one of the three biological replicates inoculated per strain. Fire lookup table (LUT) indicates fluorescent signal intensity (see insert, left panel). **(B)** Quantitative analysis of *gfp* expression on *P. vulgaris* root surface and at 3 cm distance from the root. Blue circles represent individual data points. Error bars indicate the standard error of the mean (SEM). Significant differences between samples were analyzed with one-way ANOVA with Tukey’s test (*p*-value ≤ 0.05). Same letters indicate that samples are not statistically significantly different (a–c).

To examine the expression of the *iaaMH* genes in the early stages of symbiosis, 2 day-old germinated *P. vulgaris* seeds were placed on soft-agar nitrogen-limited medium plates inoculated with *P. phymatum* STM815 wild-type and a *nifA* mutant containing the *iaaMH-gfp* reporter plasmid (hereafter WT-_*p*_*iaaMH* and *nifA*-_*p*_*iaaMH*, respectively). *P. phymatum* wild-type containing the empty pPROBE vector (WT-pPROBE) was used as negative control. As a positive control, the *P. phymatum* reporter strain _*p*_*nodB*, containing the promotor of the *nodB* gene (Bphy_7722) fused to *gfp*, was employed (WT-_*p*_*nodB*) (Hug et al., 2021). The gene *nodB* is involved in the synthesis of the backbone of NFs, and its expression is known to be induced by flavonoids secreted by the host plant (Guerreiro et al., 1997). After 3 days of incubation at 28°C, the *gfp* expression of the reporter strains co-inoculated with the germinated seeds was examined under the fluorescence microscope. While the negative control only displayed root autofluorescence, the *iaaMH-gfp* reporter in the wild-type strain was expressed at a similar level as the *nodB-gfp* fusion in the vicinity of the root apex (Figure 3A). GFP signals consistently decreased with increasing distance to the root to reach background levels at approx. 3 cm from the apex (Figure 3B). This indicates that *nodB* and *iaaMH* promotor activities depended on diffusible root exudates in our assays. In the *nifA* mutant, *iaaMH-gfp* signals were two-fold up-regulated compared to wild-type levels close to and also 3 cm away from the root apex (Figure 3B). These results suggest that NifA represses the expression of *iaaMH* and that root exudates induce *iaaMH* expression.

### *Paraburkholderia phymatum iaaMH* expression is constitutive and controlled by NifA during the establishment of *Paraburkholderia phymatum–Phaseolus vulgaris* symbiosis

**FIGURE 4:**
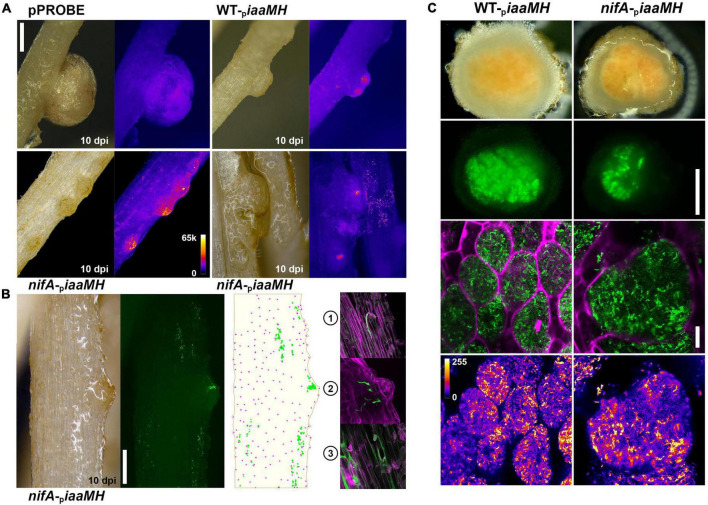
*In situ* monitoring of *iaaMH* promotor activity. **(A)**_*p*_*iaaMH*-driven *gfp* expression in *Phaseolus vulgaris* roots in the given conditions. Upper-left, note the absence of specific fluorescence in mature nodules occupied by pPROBE-only inoculants. Upper-right, wild-type *Paraburkholderia phymatum* occasionally triggers fusing nodules. Note the fluorescent signals scattered on the root epidermis. Lower left, note the higher GFP fluorescence in single and fused developing nodules or on the epidermis infected by the *nifA* mutant. Lower right, two *nifA*-occupied roots displaying either developing nodules or heavily colonized epidermal area. All micrographs were acquired with the same acquisition settings, Fire LUT, 16-bit depth. Bar = 500 μm. **(B)** GFP signals distribution on a *nifA*-colonized root. The cartoon depicts the overlapping of GFP signals (green) and identified root hair positions (magenta) from the micrograph. Bar = 500 μm. (1) An arrested IT. (2) A branching IT and nodular outgrowth. (3) ITs and densely colonized epidermal cells. **(C)** Upper panel, transversal sections of _*p*_*iaaMH*:*gfp* expressing 21 dpi mature nodules colonized by wild-type and *nifA* strains. Note the coloration indicating leghemoglobin production in both nodule types. *nifA*-_p_*iaaMH* infected cells lose more frequently the fluorescence provided by the pPROBE construct. Bar = 500 μm. Lower panel, _*p*_*iaaMH*-driven bacteroids *gfp* expression in infected cells (green). Calcofluor staining (magenta). Maximum z-projections of 10x × 0.5 μm LSCM optical sections. Lower images display the green signal quantitatively. Fire LUT, 8-bit depth. Bar = 10 μm.

To investigate in which step(s) of *P. vulgaris* nodule organogenesis the *P. phymatum iaaMH* genes are expressed and controlled by NifA, we used the reporter strains previously described to track the changes in *iaaMH* expression and bacterial colonization patterns during the different development stages of the symbiosis. Germinated seedlings of *P. vulgaris* were inoculated with the *P. phymatum iaaMH* reporter strain in a wild-type (WT-_*p*_*iaaMH*) and *nifA* mutant background (*nifA*-_*p*_*iaaMH)*. The plants were incubated for 10, 14 (during the formation of young nodules), and 21 days (optimal nitrogen fixation activity) and the GFP fluorescence signals in developing and mature nodules were microscopically monitored, either non-invasively or from transversal sections. As expected, negative control plants containing the empty pPROBE vector did not show specific GFP signals, although auto fluorescence occurred in mature nodules (Figure 4A). However, the *iaaMH* promoter was active at all tested time points during the infection process. This is congruent with the data presented in the previous section, pointing to an early activation of bacterial auxin production. Additionally, in absence of NifA, the *iaaMH* genes were expressed at greater levels compared to the wild type in all stages of nodule organogenesis, suggesting that NifA is tightly controlling auxin biosynthesis throughout the symbiosis. Although we cannot exclude that *P. phymatum* expressing low levels of *iaaMH-gfp* were present in our samples and initiated infection sites, we observed a plethora of specific GFP signals strongly associated with the plant tissues. This *gfp* expression was not due to surface-attached bacteria, as the thorough washing steps we applied to the roots easily removed such microcolonies. We could readily visualize *iaaMH-gfp* expression from nascent primordia to mature nodules in plants colonized with both strains (Figure 4A). Roots infected with WT-_*p*_*iaaMH* occasionally showed fusing infected primordia and numerous scattered GFP *punctuae*. We found this pattern strikingly enhanced on *P. vulgaris* roots inoculated with *nifA*-_*p*_*iaaMH* (Figure 4A and Supplementary Figure 3), where *iaaMH-gfp* promotor activity was regularly observed in multiple adjacent developing nodule primordia. By closely inspecting the distribution of the GFP signals outside of the nodular outgrowths, we located them in direct proximity to root hair cells (Figure 4B). Confocal microscopy established that the majority of these fluorescent signals represent *P. phymatum* ITs. We could partition these infection sites into three groups: (1) elongated ITs that failed reaching the cortical layer, (2) branching cortical ITs with emerging nodular primordia and (3) large and diffuse ITs occupying infected epidermal cells (Figure 4B). We could not determine if the colonized epidermal cells were preferentially root hairs in the latter group. At day 21 post infection, both plants colonized with WT-_*p*_*iaaMH* or *nifA*-_*p*_*iaaMH* showed nodules with leghemoglobin coloration and equivalent GFP signals in their respective infected cells (Figure 4C and Supplementary Figure 4).

### *Paraburkholderia phymatum iaaMH* genes are essential for the production of indole-acetamide and indole-3-acetic acid in *Phaseolus vulgaris* nodules

**FIGURE 5:**
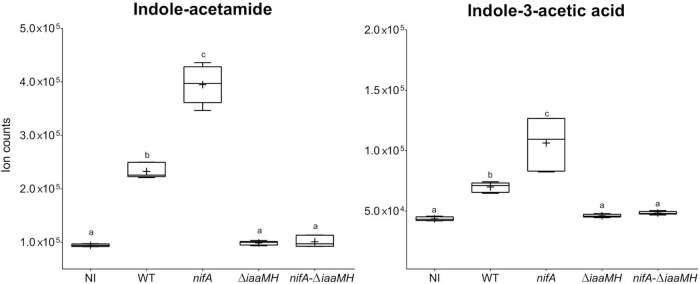
Ion counts observed for indole-acetamide (IAM) and indole-3-acetic acid (IAA). The respective levels are indicated for *Phaseolus vulgaris* non-inoculated roots (NI) and nodules occupied by *Paraburkholderia phymatum* wild-type (WT), *nifA* mutant (*nifA*), as well as Δ*iaaMH* and *nifA*-Δ*iaaMH* double mutant strains. Plus-symbols (+) indicate the arithmetic mean while whiskers represent minimum and maximum values. Differences between samples were analyzed with one-way ANOVA with Tukey’s test (*p*-value ≤ 0.05). Statistically significant differences are indicated with different letters (a–c).

The involvement of the *P. phymatum iaaMH* genes in the production of indole-acetamide (IAM) and indole-3-acetic acid (IAA) was confirmed using a metabolomics analysis on nodules infected by *P. phymatum* wild-type, the *nifA* insertional mutant, an *iaaMH* deletion mutant (Δ*iaaMH*) and a *nifA*-Δ*iaaMH* double mutant strain. Metabolites of *P. vulgaris* nodules induced by these different *P. phymatum* strains were extracted 21 days after inoculation and were compared to those extracted from uninfected roots. Three independent biological replicates inoculated per strain were analyzed using a non-targeted metabolomics approach by flow injection time-of-flight mass spectrometry (Lardi et al., 2016, 2018; Bellés-Sancho et al., 2021a,b). A total of 285 ions were matched to deprotonated metabolites and were annotated based on the accurate mass. Out of all detected metabolites, we focused on the ion counts belonging to IAM and IAA. As we previously reported, plants inoculated with *P. phymatum* wild-type produced significantly higher amounts of IAM and IAA than non-inoculated plants (Bellés-Sancho et al., 2021a), while the levels of these two auxins in the *nifA* mutant were found elevated compared to the wild-type nodules (Figure 5; Bellés-Sancho et al., 2021a). The amount of IAM and IAA in nodules induced by the single Δ*iaaMH* and double *nifA*-Δ*iaaMH* mutant decreased to the same levels as observed in non-inoculated roots, suggesting that bacterial auxin production accounted for the main portion of IAA and IAM found in wild-type and *nifA* nodules (Figure 5). We therefore conclude that the presence of the *iaaMH* genes lead to IAA and IAM production and confirm that the key regulator of nitrogen fixation, NifA, negatively regulates IAA and IAM amounts in *P. phymatum-P. vulgaris* nodules. Only few metabolites were found to be differentially abundant in nodules infected with a Δ*iaaMH* mutant. In addition to IAA and IAM, significantly lower amounts of the metabolites xanthine, linamarin, 4-phospho-L-aspartate and pantothenic acid were found in the nodules infected with the Δ*iaaMH* mutant compared to the wild-type (Supplementary Table 4). Moreover, in nodules formed by the Δ*iaaMH* mutant, the intermediate of the lysine degradation pathway saccharopine, the precursor of jasmonic acid alpha-linoleic acid, the serotonin degradation product 5-hydroxylacetic acid and 1-nitronaphthalene-5,6-oxide were detected at higher levels.

### NifA-dependent clustering *of Phaseolus vulgaris* nodules is induced by the presence of *Paraburkholderia phymatum iaaMH* genes

**FIGURE 6:**
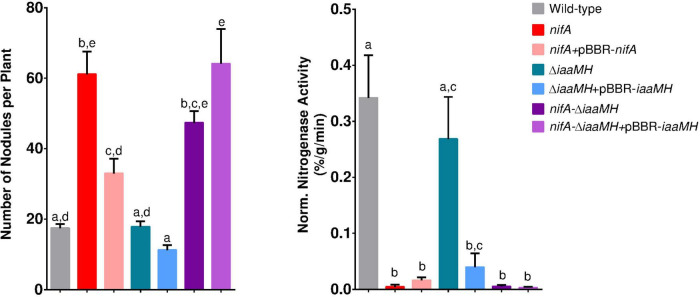
Symbiotic properties of *Paraburkholderia phymatum* wild-type, *nifA* mutant, *nifA* complemented strain, Δ*iaaMH*, Δ*iaaMH* complemented strains, *nifA*-Δ*iaaMH* double mutant and *nifA*-Δ*iaaMH* double mutant complemented with *iaaMH*. At least four biological replicates were used, consisting of a minimum of four plants per replicate. Error bars indicate the standard error of the mean (SEM). For each histogram, values with the same letter are not significantly different (a–e) (ANOVA, Tukey’s test with *p*-value ≤ 0.05).

To evaluate the possible contribution of *P. phymatum*’s auxin production in the NifA-dependent hypernodulation and nodule clustering phenotype in *P. vulgaris* (Lardi et al., 2017b; Bellés-Sancho et al., 2021a), the symbiotic properties of *P. phymatum* wild-type, *nifA* mutant, Δ*iaaMH* and the *nifA*-Δ*iaaMH* double mutant were characterized. Compared to the plants inoculated with *P. phymatum* wild-type, plants in symbiosis with Δ*iaaMH* did not show any significant difference in any of the properties tested (number of nodules, dry weight per nodule, normalized nitrogenase activity), suggesting that the absence of bacterial auxin alone did not significantly affect symbiosis with the common bean (Figure 6 and Supplementary Table 5). Moreover, the Δ*iaaMH* strain complemented with *iaaMH* expressed from its natural promoter also showed no difference in the symbiotic properties compared to the mutant strain. The hypernodulation phenotype of the *nifA* mutant was reverted to wild-type levels when the *nifA* gene was complemented on a plasmid. Similar to the phenotype observed in plants inoculated with the *nifA* mutant, the *nifA*-Δ*iaaMH* double mutant showed a significantly higher number of nodules compared to the plants inoculated with the wild-type. However, the nodule number in the double mutant was slightly reduced compared to the *nifA* mutant, although this difference was not significant (Figure 6). This suggests that bacterial auxin contributes to increase hypernodulation when NifA is absent. Like the *nifA* mutant, the *nifA*-Δ*iaaMH* mutant showed no nitrogenase activity, indicating that NifA controls expression of the nitrogenase independently of the presence of the auxin biosynthesis genes. We next determined the number of bacteroids inside nodule occupied by the wild-type and the different *nifA* and *iaaMH* mutant strains and found that the nodule occupancy was similar for each strain (Supplementary Figure 5). Remarkably, plants inoculated with Δ*iaaMH* and the *nifA*-Δ*iaaMH* double mutant complemented with *iaaMH* under control of the strong *lacZ* promotor induced abnormal, thicker roots without nodules, presumably due to an excess of exogenous auxin production (Supplementary Figure 6). These results confirm that *P. phymatum iaaMH* is not essential for a functional symbiosis with *P. vulgaris*. However, auxin seems to influence the nodulation frequency observed with the *nifA* mutant.

**FIGURE 7:**
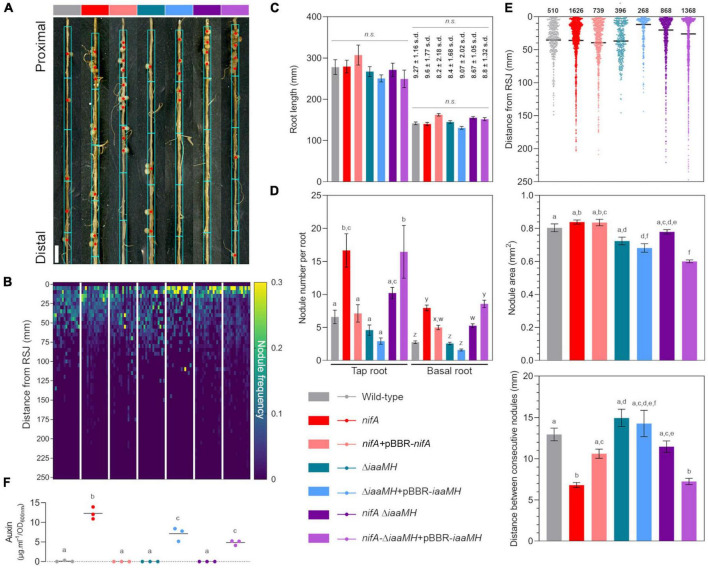
Detailed characterization of *Paraburkholderia phymatum-Phaseolus vulgaris* symbiosis nodulation patterns in the presence of discrete strains. **(A)** Representative 21 dpi *P. vulgaris* basal roots infected with the indicated strain. Red dots indicate the centroid of each single or clustered nodules. Cyan boxes illustrate the suggested nodulation windows along the root axis. Bar = 5 mm. **(B)** Frequency of nodulation in the entire root system of 15 individual plants in response to the given inoculants. The positions of individual nodules relative to the root-shoot junction were pooled into 5 mm bins. **(C)** Mean tap and basal root lengths of the sampled plants. Whiskers represent standard error. Bold numbers indicate the mean number of basal roots and standard deviation of the mean. Significant differences between samples means were analyzed with one-way ANOVA with Tukey’s *post hoc* test (*p*-value ≤ 0.05). Same letters indicate that means are not statistically significantly different when required. *n.s.*, not significant. **(D)** Mean nodule number per root type. Whiskers represent standard error. **(E)** Total nodules parameters for the sampled plants. Individual dots represent single nodule position on the root axis. Black bars represent means. Whiskers represent standard error. Bold numbers indicate the total number of sampled nodules. Significant differences between samples means were analyzed with one-way ANOVA with Tukey’s *post hoc* test (*p*-value ≤ 0.05). Same letters indicate that means are not statistically significantly different. **(F)** Quantification of indolics production in liquid cultures of the given strains in μg⋅ml^–1^ in relation to the cultures’ optical density OD_600_. Individual dots represent each different biological replicate. Different letters account for significantly different values.

To determine the impact of NifA and IaaMH functions and their interplay at a finer resolution, we conducted a precise phenotypical characterization of common bean plants 21 days post inoculation (dpi) by monitoring their root parameters in presence of different strains. We first recorded the position of each individual nodule along the axis of each tap and basal root from 15 plants. At this stage, all plants presented comparable statures and apparent health. Figure 7A shows the first centimeters of representative basal root colonization. We used this positional information to build a map of nodule frequency distribution for the entire root system of the individual samples (Figure 7B). This dataset allowed us to conclude that, irrespective to the tested bacterial strain, the bulk of *P. vulgaris* nodulation occurred within the first 75 mm of the root system, with a large portion of nodules developing in the few first centimeters of the roots. This indicated us that the changes in auxin production measured in *nifA* and *iaaMH* mutants did not dramatically influence nodule positioning along the root developmental axis. As an imbalance in auxin homeostasis has often been reported to alter root development and growth, we sought to measure the status of the root system in our experiment. The number of basal roots developed per plant were not significantly different between the tested conditions (Figure 7C). We also observed no significant differences in the lengths of tap roots or basal roots between samples, respectively (Figure 7C). Although we did not measure these parameters, the lateral root and root hair densities also appeared homogeneous in all tested plants. Thus, we found no compelling evidence of a direct bacterial auxin effect on the systemic root system architecture, indicating that its contribution to the symbiosis might be locally restricted. In contrast, the loss of NifA function nearly tripled the number of developed nodules on tap and basal roots (Figure 7D). This increase was restored to wild-type levels in plants colonized with the complemented *nifA* strain, although the number of nodules per basal root remained significantly greater than wild-type numbers. Interestingly, the Δ*iaaMH*-colonized plants displayed a nodule number per root close to wild type, even slightly lower, a tendency that seemed more pronounced in inoculations with the functionally complemented strain Δ*iaaMH* + pBBR-*iaaMH*. Finally, the inactivation of *iaaMH* in the *nifA* mutant background resulted in a clear mitigation of the *nifA* nodulation phenotype, the latter being restored in plants inoculated with a *nifA* strain harboring the complemented *iaaMH* mutant. Taken together, our data strongly suggest that NifA governs root nodular density via negative control of *iaaMH* expression in *P. phymatum.* However, by visualizing the 1D distribution of the positions of the whole population of nodules on the roots of each condition tested here, it appeared that the obtained dot plots displayed comparable shapes and local densities. We then hypothesized that the hypernodulation triggered in *nifA*-colonized plants might solely be explained by an enrichment of developed nodules in pre-defined root areas (Figure 7E). We first verified that the sizes of the nodules colonized by the various strains did not significantly differ (Figure 7E). To our surprise, the nodules occupied by the *iaaMH* mutant appeared smaller. Nevertheless, we retained a mean diameter of 1 mm for all nodules. By calculating the distance between consecutive nodules along the axis of individual roots, the clustering of *nifA* nodules was manifest (Figure 7E). This phenotype was alleviated by either the complementation of the NifA function or by disrupting IaaMH-mediated auxin synthesis in the *nifA* background. Restoring *iaaMH* functionality in the *nifA* strain led to the *nifA* phenotype. Single manipulations of the *iaaMH* genes did not influence nodule clustering when compared to roots colonized with the *P. phymatum* wild-type. We next categorized the frequency of consecutive nodules spacing into 1 mm bins (Supplementary Figure 7A) and uncovered that 46.3% of all *nifA*-occupied nodules aggregated into root sections under 3 mm in length. In comparison, nodules occupied with the *nifA* complemented strain (31.0%), Δ*iaaMH* (23.3%), Δ*iaaMH* + pBBR-*iaaMH* (36.4%), or *nifA*-Δ*iaaMH* (37.7%) showed similar clustering to wild-type-colonized nodules (21.5%) in such segments. Within this window, the nodules hosting the *nifA*-Δ*iaaMH* + pBBR-*iaaMH* strain displayed a clustering frequency of 53.8%, congruent with *nifA* values. To establish if the observed nodular clusters were indiscriminately scattered onto the root axis, we therefore subjected our samples’ 1D root nodular coordinates to Jenks natural breaks optimization (Supplementary Figure 7B). Our computations broke the different datasets into 10 classes with goodness of variance fits over 98%. We deduced from the classes limits the root windows in which nodulation occurred for all tested conditions. Regardless of the inoculants and the number of nodules, *P. vulgaris* nodulation occurred on roots in discrete windows consistently increasing in size while progressing toward the root apex, a pattern remarkably reminiscent of the lateral root emergence developmental process. Within the first 60–75 mm of roots, all inoculated plants displayed a uniform windowing ranging from 10 to 20 mm, approximatively (blue boxes in Figure 7A). In younger parts of the roots, nearer the apices, the nodules of roots colonized by the *nifA*, *nifA* complemented, *nifA*-Δ*iaaMH* and *nifA*-Δ*iaaMH* + pBBR-*iaaMH* strains displayed larger nodulation windows. Consistent with the rest of our data, plants hosting wild-type, Δ*iaaMH* or Δ*iaaMH* + pBBR-*iaaMH* shared very similar distributions. In comparison, a population of randomized values were evenly distributed in 10 windows of about 20 mm along a theoretical root axis of 250 mm.

Taken together, our data suggest that the clustered hypernodulation observed in *nifA*-colonized plants results from a strong increase in developed nodules originating from discrete, facilitating root sites. In analogy to lateral root development, such sites might be genetically or hormonally primed to respond to internal auxin signaling. To confirm that the nodular levels of auxinics we previously measured (Bellés-Sancho et al., 2021a) originated from bacterial production under NifA-IaaMH regulation, we estimated the amount of indolics in our strains after planktonic growth (Figure 7F). In comparison to wild-type *P. phymatum* that produced traces of indolics, the *nifA* mutant synthetized considerable amounts (37.9 μg/ml). This trait was returned to wild-type levels in the *nifA*-complemented and *nifA*-Δ*iaaMH* strains. As expected, the Δ*iaaMH* was unable to produce auxinics. However, we found that the presence of pBBR-*iaaMH* in either the Δ*iaaMH* or *nifA*-Δ*iaaMH* backgrounds displayed similar elevated levels of auxinics, thus implying that the production of IAA in these strains is not entirely controlled by NifA. This might, at least partially, explain the lower nodule number observed in plants hosting Δ*iaaMH* + pBBR-*iaaMH* and the smaller nodule sizes triggered by strains expressing the pBBR-*iaaMH* plasmid. We conclude that the *P. phymatum* IaaMH-dependent auxin synthesis is directly involved into the initiation and development of *P. vulgaris* nodules in predetermined susceptible zones along the root axis.

### The *iaaH and iaaM* genes are present in the genus *Paraburkholderia* and are related to auxin production genes from phytopathogenic species

**FIGURE 8:**
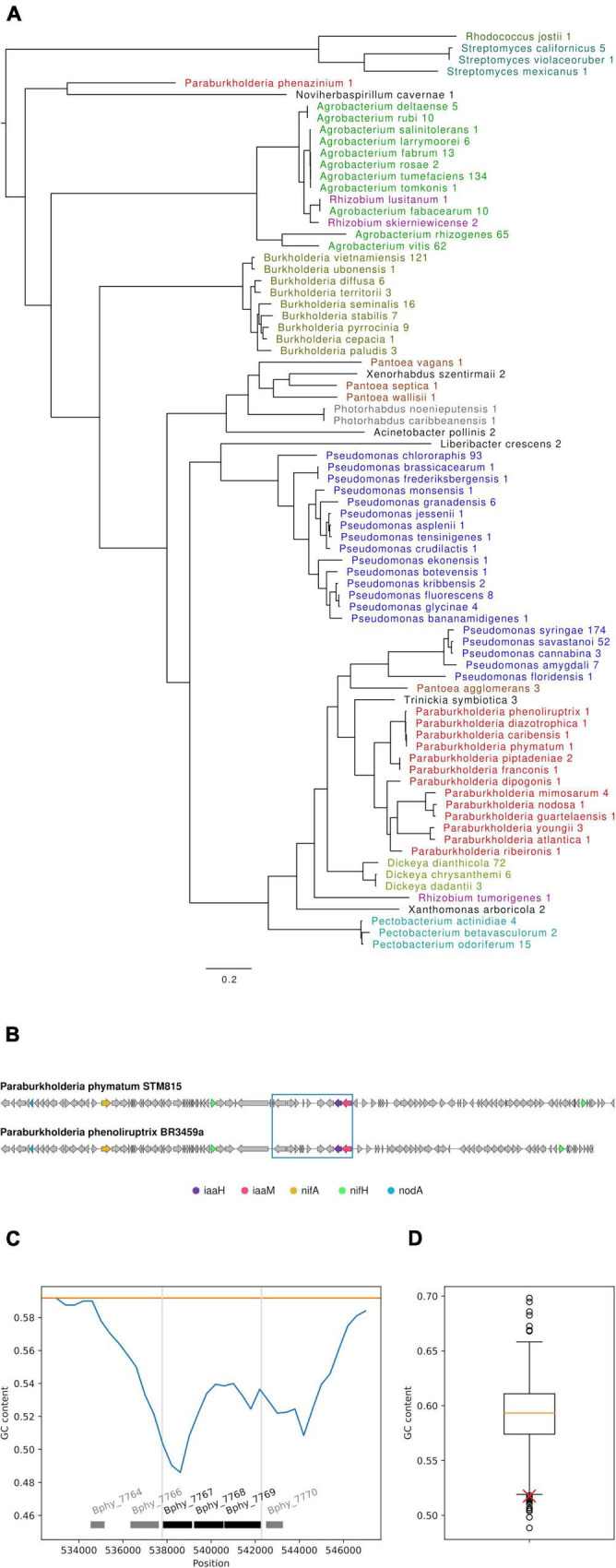
*In silico* analysis of *Paraburkholderia phymatum iaaMH* genes. **(A)** Maximum likelihood phylogenetic tree of co-occuring orthologs of IaaH and IaaM in other species identified by cblaster. The numbers indicate how many strains per species were found. When more than one strain was identified for a species, only the genome best representing the species was chosen **(B)** Synteny of *iaaM* (pink), *iaaH* (purple), *nifA* (yellow), *nifH* (green), and *nodA* (blue) between *P. phymatum* STM815 (top) and *P. phenolirupterix* BR3459a (bottom). Note that in both strains, two copies of *nifH* are present. A BLAST of the DNA sequence from Bphy_7758 to Bphy_7769 (blue box) identified *P. phenoliruptrix* BR3459a as the only other strain containing the whole sequence (100% sequence coverage of 13,791 nucleotides, 99.4% nucleotide identity). **(C)** GC content (blue line) of the *P. phymatum iaaMH* genes and the surrounding genomic region on the symbiotic plasmid, analyzed with a sliding window analysis (see “Bioinformatic analysis” section in “Materials and methods”). The mean GC content of the plasmid is shown as orange line, the extent of the predicted auxin operon is shown by gray vertical lines and the genes in black. A potential IS5 family transposase gene (Bphy_7770) is located to the right side of *iaaM*. **(D)** Boxplot showing the GC content distribution of all windows of similar size on the symbiotic plasmid (NC_010627.1). The GC content of the window containing the three predicted auxin operon genes (red cross) shows a significantly lower GC content.

To identify orthologs of *P. phymatum* STM815’s *iaaMH* genes in other bacterial species, an *in silico* analysis using the respective *P. phymatum* protein sequences as query for a cblaster search was carried out (see section “Bioinformatic analysis” in “Materials and methods”). Strains retrieved from this analysis were required to have both orthologs located adjacent to each other and to have a full species name (sp. hits were not allowed as they might bias the results). Among the 1,001 strains satisfying these criteria, we found 174 *Pseudomonas syringae*, 134 *Agrobacterium tumefaciens*, 121 *Burkholderia vietnamensis*, 93 *Pseudomonas chloroaphis*, 72 *Dickeya dicanthola*, 65 *Agrobacterium rhizogenes*, and 62 *Agrobacterium vitis* strains. These top seven species accounted for more than 70% of all hits. Overall, the strains belong to 79 different species many of which were represented by a single strain, including several *Paraburkholderia* species (Supplementary Table 3). To create a phylogenetic tree, we selected one representative strain per species, preferentially including NCBIs representative genome, reference genomes or complete genomes over fragmented Illumina assemblies (see section “Bioinformatic analysis” in “Materials and methods”). The phylogenetic analysis revealed several distinct groups of strains containing IaaM and IaaH (Figure 8A). In order to investigate potential functional links between proteins related to auxin production with other rhizobial traits (such as nitrogen fixation and nodulation) at a genomic level, we analyzed the co-occurrence of *nifA, nifH* and *nodA* genes in bacterial strains that encode the *iaaMH* genes (Supplementary Table 3). Orthologs of all five genes were only present in 30 strains belonging to several species from the genus *Paraburkholderia*, namely *P. phenoliruptix, P. ribeironis, P. atlantica, P. youngii, P. dipogonis, P. nodosa, P. guartelaensis, P. mimosarum, P. caribensis*, *P. diazotrophica, P. franconis, P. piptadeniae* and *P. phenazinium*, and three strains of *Trinickia symbiotica.* Notably, *P. phenoliruptix* was the strain with the highest homology score for all five gene products present on the same contig (minimum and maximum score 3.21 and 8.48, respectively). The homologous region in this strain showed 100% sequence coverage and 99.4% nucleotide identity with the one of *P. phymatum* (Figure 8B). Three strains belonging to *Dickeya* genus (*D. chrysanthemi, D. dianthicola*, and *D. dadantii*) presented, in addition to *iaaMH*, the *nifA* and *nifH* genes. Among the strains that only carried orthologs of the *P. phymatum iaaMH* genes (without *nif* or *nod* genes), high similarity was found in species belonging to the genera *Agrobacterium, Pantoea, Pseudomonas, Rhizobium, Xanthomonas, Pectobacterium, Acinetobacter, Photorhabdus, Xenorhabdus, Liberibacter, Noviherbaspirillum, Streptomyces*, and *Rhodococcus.* Outside of the *Paraburkholderia* group, the IaaMH orthologs of the phytopathogenic strains *A. vitis*, *A. rhizogenes*, and *A. tumefaciens* showed the highest similarity with the *P. phymatum* genes. Orthologs of *P. phymatum* IaaMH (maximum and minimum score 3.08) were also found in *Burkholderia* strains belonging to the Burkholderia cepacia complex (Bcc) that are opportunistic human pathogens, with the addition of *B. vietnamiensis* and *B. ubonensis* which also carry *nif* genes (Eberl and Vandamme, 2016). The region containing the *P. phymatum iaa* operon is located on the symbiotic plasmid and showed a significantly lower GC content (a difference of 0.074) compared to the rest of the plasmid (0.592) (Figures 8C,D). Furthermore, our bioinformatic analysis predicted that Bphy_7770 (the gene upstream of *iaaM*), codes for a putative IS5 family transposase. This result together with the phylogenetic reconstruction analyses suggests that the *iaaMH* genes in *P. phymatum* and other *Paraburkholderia* strains may have been acquired horizontally from a phytopathogenic ancestor.

## Discussion

We present here the first study of the nodule architecture and organization induced by a beta-rhizobial *nifA* mutant and associate it to beta-rhizobial auxin synthesis during symbiosis with *P. vulgaris*.

As reported earlier (Bellés-Sancho et al., 2021a), the *P. phymatum nifA* mutant triggers nodule clustering (Figures 1A,B). Other than this phenotype, no abnormalities were found in *nifA* nodules (Figures 1C–F) or root traits. We reasoned that this phenotype might originate from the deregulation of early nodule proliferation, presumably from primary cortical initiation sites. Plant cells infected with the *nifA* mutant were found larger than the ones infected with the wild type (Figure 2B), a subtle change that we can attribute to the effects of auxin, as the hormone drives cell elongation (Majda and Robert, 2018). No difference was observed between wild-type and *nifA* bacteroids (Figure 2A), which is in contrast to reports showing that the lack of NifA provokes bacteroid death in *S. meliloti* 1021-*M. truncatula* symbiosis (Berrabah et al., 2015) seemingly sanctioning inefficient Fix^–^ symbionts via a plant immunity response.

Interestingly, in alpha-rhizobia, NifA was reported to positively control the production of rhizobial hormones (Nukui et al., 2006; Murset et al., 2012; Nett et al., 2022). For instance, NifA in *B. diazoefficiens* USDA 110 has been shown to control the synthesis of gibberellin, a plant hormone also produced by bacteria, which induced bigger nodules in soybean in later stages of the symbiosis (Nett et al., 2022). We showed here that during the early phase of the *P. phymatum-P. vulgaris* symbiosis, the bacterial *iaaMH* genes are induced by root exudates (Figure 3). In line with this observation, a previous transcriptomics analysis had shown that *P. phymatum iaaMH* expression was 1.8-fold upregulated when exposed to *Mimosa pudica* root exudates (Klonowska et al., 2018). Root exudates are composed of a blend of primary and secondary metabolites, including the amino acid TRP, a precursor of IAA (Spaepen et al., 2007; Vives-Peris et al., 2020). In other strains, TRP induces the expression of IAA biosynthetic genes from either the IPA pathway in *Enterobacter cloacae* UW5 (Ryu and Patten, 2008) or the IAM pathway in *P. syringae* pv. *syringae* and *D. dadantii* 3937 (Duca and Glick, 2020). Flavonoids are also compounds excreted by the roots, which can induce the expression of genes related to IAA synthesis, as shown in *Rhizobium* sp. NGR234, where the IPA pathway is induced by the transcriptional regulator NodD (Theunis et al., 2004). In addition, flavonoids not only influence bacterial auxin production, but can also inhibit plant auxin transport in roots during nodule formation, leading to auxin accumulation in the infection zone (Mathesius et al., 1998; Brown et al., 2001; Wasson et al., 2006; Peer and Murphy, 2007; Breakspear et al., 2014). A previous transcriptome analysis performed by our group had shown that the expression of the *iaaHM* operon, at the time annotated as an amine oxidase, was upregulated in *P. vulgaris* nodules compared to free-living conditions (Lardi et al., 2017b), which would suggest a possible role during symbiosis. However, in this study no obvious symbiotic role of IAA and IAM were observed (Figure 6). We cannot exclude that the absence of a symbiotic phenotype is related to the plant host and the type of nodules (determinate or indeterminate) the plant forms, since legumes use auxin to achieve the initial buildup in different layers of the cortex (Kohlen et al., 2018). For instance, in studies where the bacterial auxin balance was disrupted by overproducing IAA in *R. leguminosarum* RD20, no difference was observed in determinate *P. vulgaris* nodules compared to wild type, while in *Vicia sativa* and *M. truncatula* (both forming indeterminate nodules) less nodules were formed by this strain (Pii et al., 2007; Camerini et al., 2008). A future characterization of the *P. phymatum iaaMH* mutant using other plant hosts that form indeterminate nodules will help to further decipher the role of rhizobial auxin in distinct nodule types. In addition, it is important to mention that auxin induces different effects depending on concentration and plant sensitivity toward auxin, *i.e.*, concentrations outside the optimal range lead to an inhibition of plant growth (Persello-Cartieaux et al., 2003; Remans et al., 2008). Noteworthy, abnormal root development and no nodule formation were observed when plants were inoculated with a strain artificially over-expressing *iaaMH* (Supplementary Figure 6).

Although the *P. phymatum iaaMH* mutant was not impaired in its symbiotic abilities, the overexpression of the *iaaMH* genes in a *nifA* mutant was responsible for the formation of clusters of nodules in adjoining nodule initiation sites on the plant root (Figures 1, 7). This phenotype might arise from the increased number of ITs in roots infected by a *nifA* mutant compared to the wild type (Figure 4B and Supplementary Figure 3). In line with the current hypothesis that auxin positively regulates epidermal infections (Velandia et al., 2022), auxins overexpression in the *P. phymatum nifA* mutant seems to act locally and promote the occurrence of infection events. This is reminiscent of the *L. japonicum-M. loti* symbiosis, where auxin was proposed to be a promotor of the initiation or elongation of the IT formation. In fact, the inhibition of the plant TAR-YUC auxin biosynthesis pathway impaired the IT elongation (Nadzieja et al., 2018). In *M. truncatula* infected with *S. meliloti*, it has been shown that a local auxin accumulation correlates with nodule numbers (Roy et al., 2017; Kohlen et al., 2018). In fact, the *M. truncatula* supernodulation mutant *sunn* (supernumerary nodules, an AON mutant) (Schnabel et al., 2005), showed an increased level of local auxin transport and expression of the auxin response gene *GH3* at the zone of nodule initiation (van Noorden et al., 2006, 2007). Nevertheless, we suggest that *P. phymatum* NifA activity mainly interplays with the *P. vulgaris* AON system, finally leading to a nodule number increase in predetermined root zones. In addition, the overproduction of bacterial auxin when NifA is absent is rather altering colonization frequency than being essential for nodulation. The induced infectivity driven by auxin could be associated with an increased competitive fitness of the strain, since *P. phymatum* is known to outcompete other *Paraburkholderia* species in nodulating common bean (Lardi et al., 2017a).

Interestingly, the *P. phymatum iaa* operon shares a high degree of homology with the *iaa* operon of phytopathogens (Figure 8 and Supplementary Table 3) such as *Agrobacterium tumefaciens* Q15/94 and *P. syringae* pv. *savastanoi.* While *A. tumefaciens* uses the IAM pathway as a virulence factor to induce crown gall tumors in plants (Zupan et al., 2000), in *P. syringae* IAA induces virulence (Djami-Tchatchou et al., 2020, 2022) and in *P. savastanoi* the *iaaMH* genes are involved in the colonization process of *Mytus communis* (Schiff et al., 2019). Orthologs of the *P. phymatum* STM815 *iaaMH* genes were also present in *Burkholderia* species belonging to the Bcc group, which includes human opportunistic pathogens (Supplementary Table 3). In *P. phymatum* and other nitrogen-fixing and nodulating *Paraburkholderia* strains, in addition to the close related *Trinickia symbiotica* but not in alpha-rhizobia, the *iaaM* and *iaaH* genes co-occur with nitrogen fixation (*nif*) and nodulation (*nod*) genes (Figure 8A and Supplementary Table 3), suggesting that the IAA-production could be a specific trait of plant-colonizing *Paraburkholderia* strains. The only exception was the endophytic *P. phenazinium*, which only presented the *iaaMH* (Sexton et al., 2020). Based on our phylogenetic analysis (Figure 8A) and the low GC content of the predicted *P. phymatum iaa* operon compared to the GC content average of the symbiotic plasmid (Figures 8C,D), the *iaaMH* from *Paraburkholderia* may have been horizontally acquired from a common phytopathogen ancestor. A common evolutionary origin of these genes in *A. tumefaciens*, *A. rhizogenes*, *P. savastanoi*, and *P. agglomerans* pv. *gypsophilae* has already been suggested, as well as their horizontal transfer between *P. savastanoi* pv. *savastanoi* NCPPB 3335, *P. syringae* pv. *aceris* M302273PT and *P. syringae* pv. *syringae* B728a (Morris, 1995; Patten et al., 2013; Aragón et al., 2014). Moreover, in the plant-associated bacterium *P. agglomerans* pv. *gypsophilae*, which causes opportunistic human infections, the *iaaMH* genes are located on a pathogenicity island with other virulence genes (Barash and Manulis-Sasson, 2009). Further analysis on whether the *iaaMH* operon is also associated with pathogenicity islands in other pathogenic species will provide valuable information on the evolution of these genes as virulence factors.

In summary, our data suggest that under the optimal control of NifA, IaaMH-driven auxin production might contribute to the successful establishment and progression of nodular primordia in response to cortical IT invasion. In absence of *nifA*, the unrestricted, exogenous bacterial auxin levels might intensify root hair infection and bypass the developmental check-points normally imposed by the host. This concept is supported by the extended symbiotic defects observed in plants inoculated with our *iaaMH* complementation strains. Circumventing the NifA fine tuning of bacterial auxin input in these strains presumably led to an improper homeostasis of the phytohormone and triggered root swellings, underdevelopment and poor nodulation frequency. Exploring the molecular mechanisms behind the mode of action of NifA as a negative regulator and the possible role of bacterial auxin as a symbiotic signal could grant deeper insights into the role of auxin biosynthesis in nitrogen-fixing and nodulating *Paraburkholderia* strains and will provide valuable information to improve competitiveness of rhizobia in the soil. We envision the potential pathogenic origin of the *iaaMH* genes as an evolutionary advantage trait used by root-colonizers to promote infection events in early symbiosis.

## Data availability statement

The datasets presented in this study can be found in online repositories. The names of the repository/repositories and accession number(s) can be found in the article/Supplementary material.

## Author contributions

PB-S, BH, CHA, AB, and GP contributed to conception and design the experiments. PB-S, YL, and AB performed the experiments. PB-S, BH, ES, CHA, LE, NZ, AB, and GP analyzed the data. PB-S, CHA, AB, and GP wrote the manuscript. All authors contributed to manuscript revision, read, and approved the submitted version.

## Footnotes

1
https://imagej.nih.gov

2
https://burkholderia.com/
