# Supplementary material for: A novel function of the key nitrogen-fixation activator NifA in beta-rhizobia: Repression of bacterial auxin synthesis during symbiosis
Source: Front Plant Sci. 2022 Sep 28;13:991548. doi: 10.3389/fpls.2022.991548 (PMC9554594; doi:10.3389/fpls.2022.991548)

Supplementary Material

**Supplementary Figure 1:** Indole-3-acetic acid (IAA) biosynthesis pathways in bacteria. The two most common TRP-dependent IAA synthesis pathway in bacteria: the indole-3-acetamide (IAM) and the indole-3-pyruvate (IPA) pathway. Each pathway is named after its main intermediate (marked in bold) and depicted with a different color.


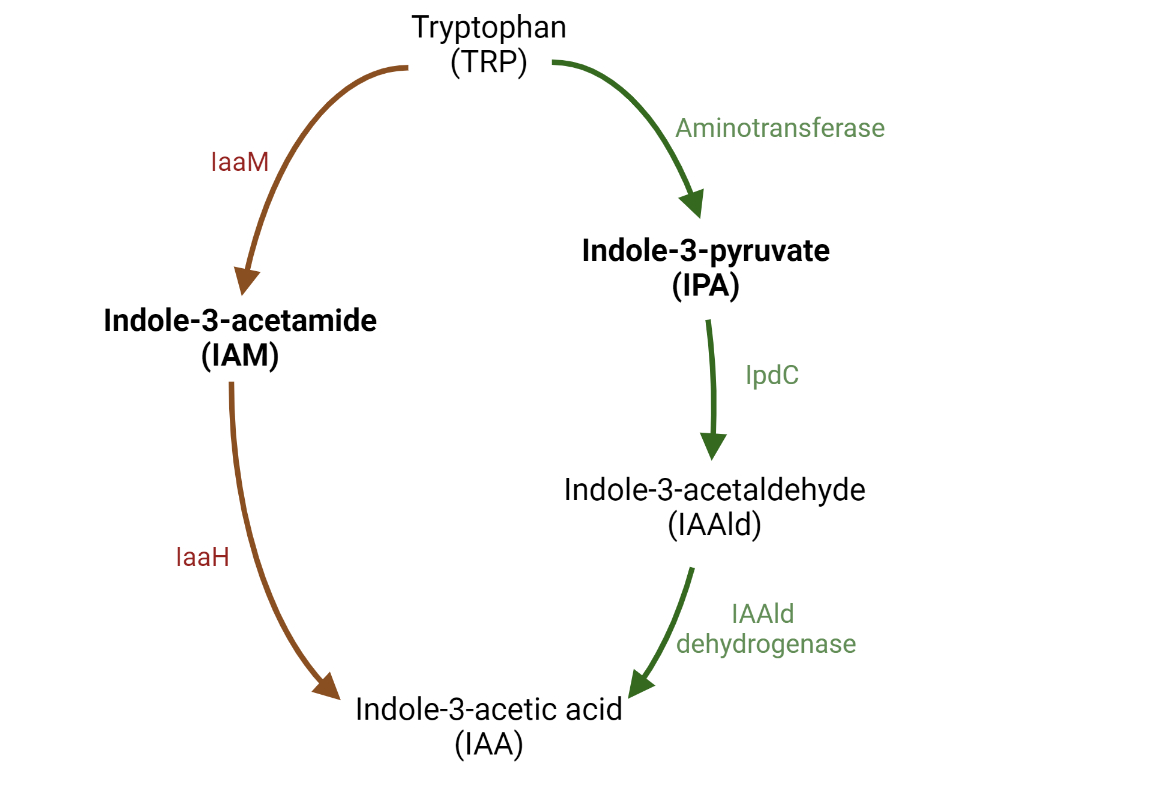


**Supplementary Figure 2:** Quantifications of live/dead staining in infected *P. vulgaris* cells. Individual infected plant cells from 3 nodules from 2 plants were assessed. Mid-section optical planes of intact cells were used, using the Calcofluor White-stained cell wall boundaries as ROIs. All signals were acquired with the same LSCM acquisition settings and propidium iodide and Syto9 specific fluorescence recorded. Mean grey values were computed in Fiji and the percentage (%) of live cells was calculated as in (Robertson et al., 2019) using equation (2). Mean % live cells and sample numbers are 58.88% n=212 for wild-type (wt), 63.44% n=207 for *nifA* mutant (nifA) and 64.73% n=221 for *nifA*+pBBR-*nifA* (nifA_compl). Significant different means are indicated (*** p<0.001, ns, not significant; ANOVA with Tukey post-hoc test).

**
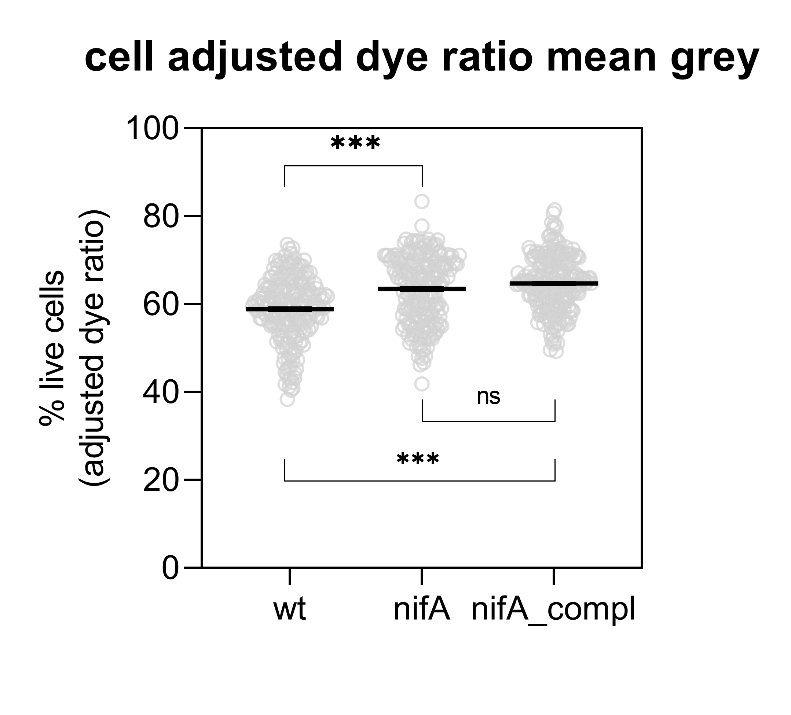
**

**Reference:**

Robertson, J., McGoverin, C., Vanholsbeeck, F., and Swift, S. (2019). Optimisation of the protocol for the LIVE/DEAD^®^ BacLight^TM^ bacterial viability kit for rapid determination of bacterial load. *Front Microbiol* 10. doi: 10.3389/fmicb.2019.00801.

**Supplementary Figure 3:** Quantifications of WT-_p_PROBE (pPROBE empty), WT-_p_*iaaMH* (wt) and *nifA-*_p_*iaaMH* (nifA) GFP signals in infected *P. vulgaris* trichoblasts. Left, mean number and mean fluorescence of *P. phymatum*-infected root hair on *P. vulgaris* inoculated roots. Three 10 dpi plants per bacterial inoculant were thoroughly washed thrice with sterile tap water and assayed. Roots were severed as 8 cm sections from the root-shoot junction and further sectioned in 2-3 cm segments. At least 15 segments (3 for WT-_p_PROBE) per plant were randomly chosen and put under the microscope. One multifocus image (1.3 x 1 µm) was obtained per segment, avoiding mature nodules and lateral roots, and analyzed using FiJi. GFP signal intensities punctuae were measured after maxima-threshold (tolerance >200, strict, exclude edge). Right, representative pictures of the root segments. All pictures were acquired with the same acquisition settings (Fire LUT). Significant differences are indicated (****p<0.0001, two-tailed unpaired t-test with Welch’s correction).

**
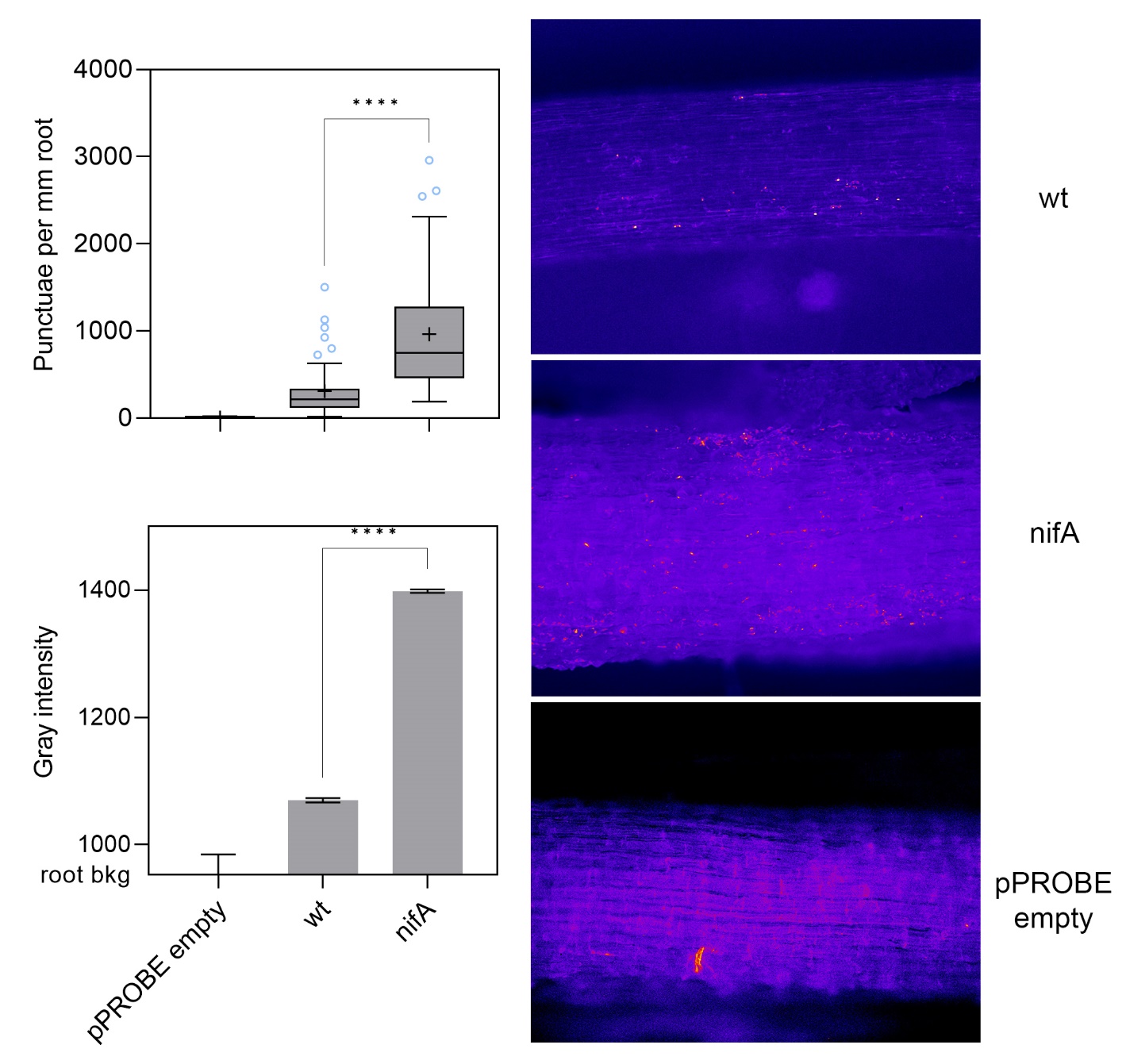
**

**Supplementary Figure 4:** Quantifications of WT-_p_*iaaMH* (wt) and *nifA-*_p_*iaaMH* (nifA) GFP signals in infected *P. vulgaris* cells. Individual infected plant cells from 3 nodules from 3 plants were assessed. Mid-section optical planes of intact cells were used, using the Calcofluor White-stained cell wall boundaries as ROIs. All signals were acquired with the same LSCM acquisition settings and GFP specific fluorescence recorded. Mean grey values were computed in Fiji. Means were found not significantly different according to two-tailed unpaired t-test with Welch’s correction (n=89 for wild-type, n=100 for *nifA*).

**
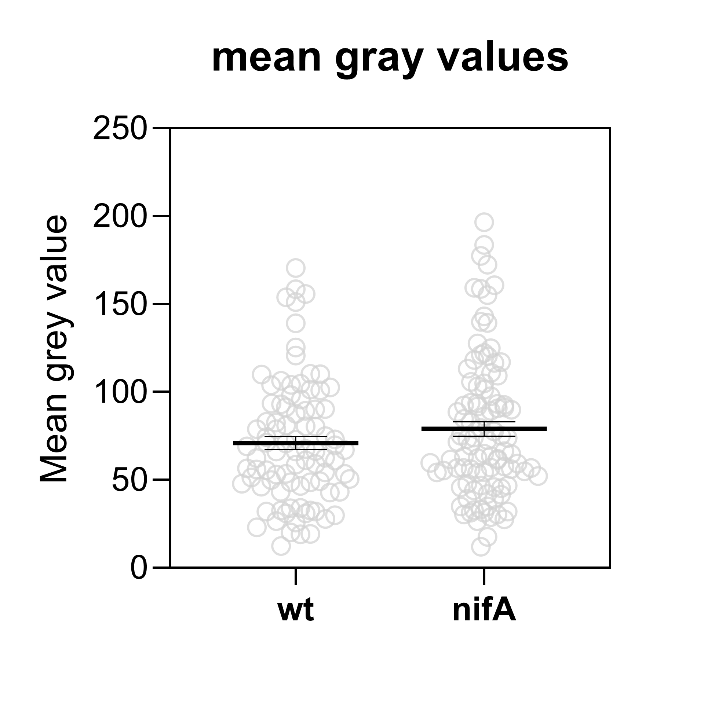
**

**Supplementary Figure 5:** Colony-forming units (CFU) re-isolated after 21 days from *P. vulgaris* nodules inoculated with *P. phymatum* wild-type (WT), *nifA* mutant (*nifA*), *nifA* mutant complemented (*nifA*+pBBR-*nifA*), *iaaMH* deletion mutant (∆*iaaMH*), *iaaMH* deletion mutant complemented (∆*iaaMH*+pBBR-*iaaMH*), *nifA-*∆*iaaMH* double mutant (*nifA-*∆*iaaMH*) and *nifA-iaaMH* mutant with *iaaMH* complemented (*nifA-*∆*iaaMH*+pBBR-*iaaMH*).


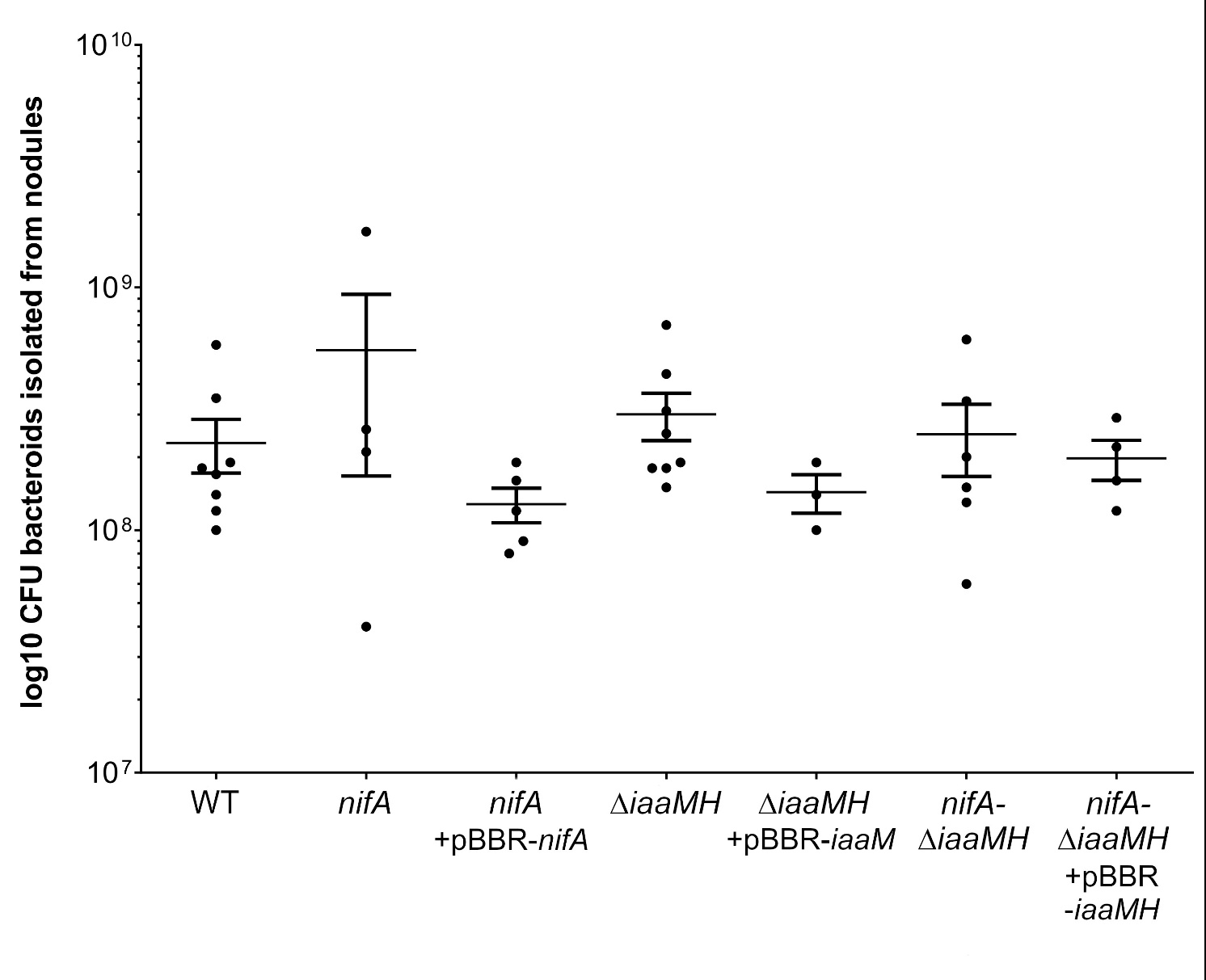


**Supplementary Figure 6:** Twenty-one days old *P. vulgaris* roots inoculated with *P. phymatum* wild-type, ∆*iaaMH* complemented in forward (∆iaaMH+pBBR-*iaaMH (*_p_*lacZ)*) and *nifA-*∆*iaaMH* double mutant *iaaMH* complemented in forward (*nifA-∆iaaMH+*pBBR-*iaaMH (*_p_*lacZ)*).

**
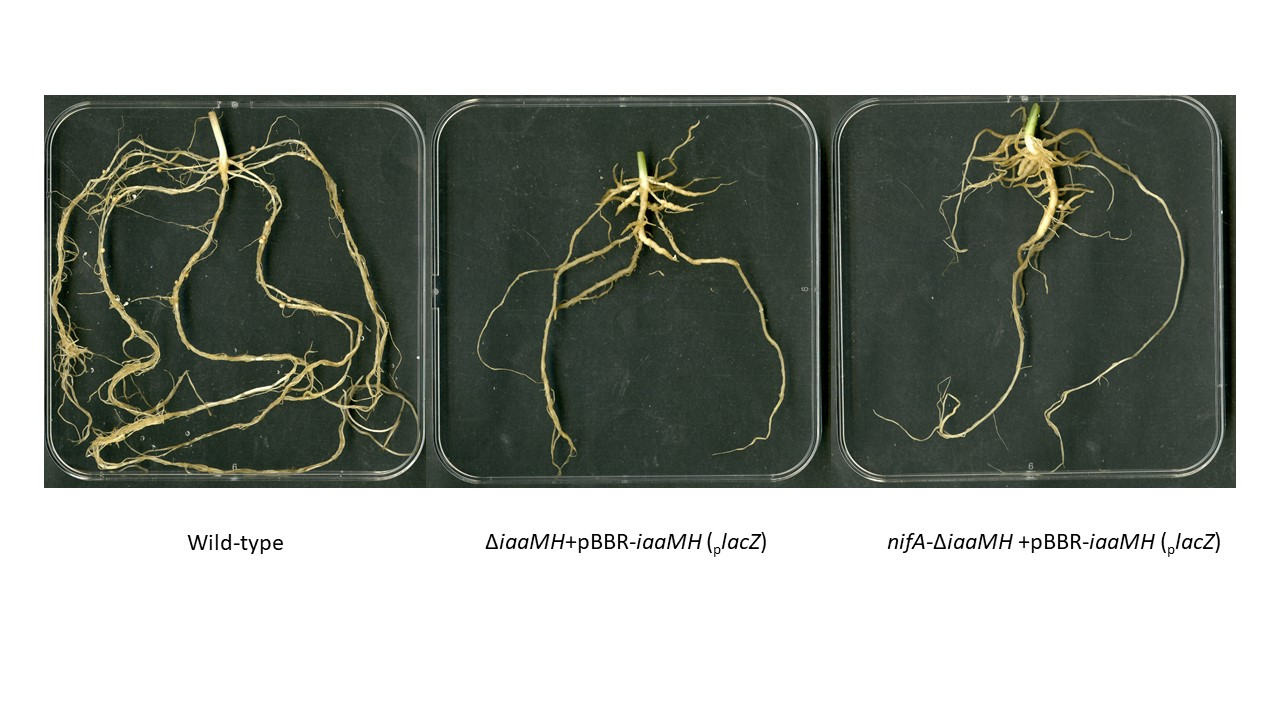
**

**Supplementary Figure 7:** Characterization of root-nodule patterns in *P. phymatum-P. vulgaris* symbiosis. (**A**) Cumulative frequencies of the distances between consecutive nodules. Each curve displays the data of one group of mutant and its respective complemented strains and wild-type data for reference. Points represent 1 mm bins. (**B**) Jenks natural breaks optimization of the dataset displayed in Figure 7E


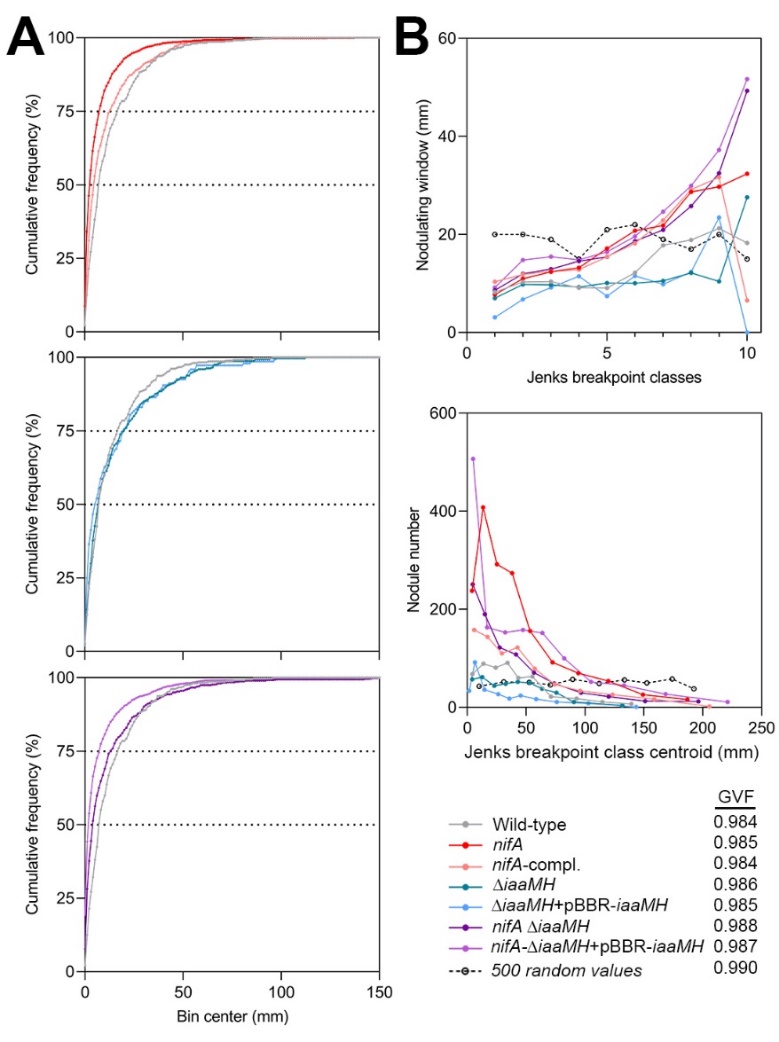

Supplement: Supplementary file 1 [file Data_Sheet_1.docx]
