# Supplementary material for: A novel function of the key nitrogen-fixation activator NifA in beta-rhizobia: Repression of bacterial auxin synthesis during symbiosis
Source: Front Plant Sci. 2022 Sep 28;13:991548. doi: 10.3389/fpls.2022.991548 (PMC9554594; doi:10.3389/fpls.2022.991548)
Supplement: Supplementary file 4 [file Table_3.docx]

**Supplementary Table 3.** Co-localization analysis of the predicted *iaaMH* operon with the *nifA*, *nifH* and *nodA* genes based on their protein sequences using cblaster. Genes were first required to be on the on the same contig such that they can be grouped into a cluster (colored background) but in a reanalysis of the cblaster results genes were also allowed on different contigs, therefore not belonging to a cluster (gray background, strains with additional genes identified are marked with an asterisk *). The cluster min. and max. columns denote the range of scores given to the clusters by cblaster based on protein conservation and positional arrangement of the genes. The respective gene min and max scores specify the protein similarity range in the given species, where similarity is the product of sequence coverage and identity (i.e., a value of 1 denotes an identical protein sequence).

|  | Protein with high similarity that is part of a cluster | | | | | | | |  |  |  |  |  |  |  |  |
| --- | --- | --- | --- | --- | --- | --- | --- | --- | --- | --- | --- | --- | --- | --- | --- | --- |
|  | Protein with low similarity that is part of a cluster | | | | | | | |  |  |  |  |  |  |  |  |
|  | Protein that is not part of a cluster (different contig or distance > 600kb) | | | | | | | |  |  |  |  |  |  |  |  |
|  |  | | | | | | | |  |  |  |  |  |  |  |  |
| Organism | | | | Cluster | | IaaM | | IaaH | | | NifA | | NifH | | NodA | |
| Genus | | Species | Strains | Min | Max | Min | Max | Min | | Max | Min | Max | Min | Max | Min | Max |
| *Paraburkholderia* | | *phymatum* | 2 | 3.21 | 8.48 | 1.00 | 1.00 | 1.00 | | 1.00 | 1.00 | 1.00 | 1.00 | 1.00 | 1.00 | 1.00 |
|  | | *phenoliruptrix* | 2 | 3.21 | 8.48 | 0.99 | 0.99 | 0.99 | | 0.99 | 1.00 | 1.00 | 1.00 | 1.00 | 0.96 | 0.96 |
|  | | *ribeironis* | 1 | 7.35 | 7.35 | 0.83 | 0.83 | 0.79 | | 0.79 | 0.82 | 0.82 | 0.98 | 0.98 | 0.89 | 0.89 |
|  | | *atlantica* | 7 | 5.27 | 7.33 | 0.78 | 0.78 | 0.72 | | 0.72 | 0.73 | 0.74 | 0.98 | 0.98 | 0.81 | 0.82 |
|  | | *youngii* | 5 | 3.16 | 7.33 | 0.78 | 0.80 | 0.72 | | 0.74 | 0.73 | 0.74 | 0.98 | 0.98 | 0.82 | 0.83 |
|  | | *dipogonis* | 1 | 7.30 | 7.30 | 0.84 | 0.84 | 0.80 | | 0.80 | 0.43 | 0.43 | 0.90 | 0.90 | 0.74 | 0.74 |
|  | | *nodosa* | 2 | 5.28 | 6.33 | 0.80 | 0.80 | 0.74 | | 0.74 | 0.74 | 0.75 | 0.95 | 0.95 | 0.85 | 0.85 |
|  | | *guartelaensis ** | 1 | 5.28 | 5.28 | 0.80 | 0.80 | 0.75 | | 0.75 | 0.74 | 0.74 | 0.95 | 0.95 | 0.84 | 0.84 |
|  | | *mimosarum ** | 4 | 5.28 | 5.28 | 0.80 | 0.80 | 0.73 | | 0.74 | 0.74 | 0.74 | 0.96 | 0.96 | 0.83 | 0.83 |
|  | | *caribensis ** | 1 | 3.21 | 3.21 | 1.00 | 1.00 | 1.00 | | 1.00 | 1.00 | 1.00 | 1.00 | 1.00 | 1.00 | 1.00 |
|  | | *diazotrophica ** | 1 | 3.21 | 3.21 | 0.99 | 0.99 | 1.00 | | 1.00 | 0.99 | 0.99 | 1.00 | 1.00 | 0.96 | 0.96 |
|  | | *franconis ** | 1 | 3.19 | 3.19 | 0.91 | 0.91 | 0.91 | | 0.91 | 0.90 | 0.90 | 0.99 | 0.99 | 0.95 | 0.95 |
|  | | *piptadeniae ** | 2 | 3.18 | 3.19 | 0.91 | 0.91 | 0.84 | | 0.91 | 0.90 | 0.90 | 0.99 | 0.99 | 0.95 | 0.95 |
|  | | *phenazinium* | 1 | 3.07 | 3.07 | 0.48 | 0.48 | 0.33 | | 0.33 |  |  |  |  |  |  |
| *Agrobacterium* | | *vitis* | 62 | 3.07 | 7.16 | 0.45 | 0.51 | 0.31 | | 0.59 |  |  |  |  |  |  |
|  | | *rhizogenes* | 65 | 3.08 | 7.16 | 0.49 | 0.51 | 0.31 | | 0.35 |  |  |  |  |  |  |
|  | | *tumefaciens* | 134 | 3.08 | 6.21 | 0.49 | 0.70 | 0.30 | | 0.61 |  |  |  |  |  |  |
|  | | *fabrum* | 13 | 3.08 | 4.12 | 0.49 | 0.51 | 0.32 | | 0.35 |  |  |  |  |  |  |
|  | | *larrymoorei* | 6 | 3.08 | 3.08 | 0.49 | 0.51 | 0.30 | | 0.34 |  |  |  |  |  |  |
|  | | *fabacearum* | 10 | 3.08 | 3.08 | 0.49 | 0.51 | 0.27 | | 0.35 |  |  |  |  |  |  |
|  | | *rubi* | 10 | 3.08 | 3.08 | 0.50 | 0.51 | 0.31 | | 0.32 |  |  |  |  |  |  |
|  | | *deltaense* | 5 | 3.08 | 3.08 | 0.50 | 0.51 | 0.31 | | 0.32 |  |  |  |  |  |  |
|  | | *rosae* | 2 | 3.08 | 3.08 | 0.51 | 0.51 | 0.32 | | 0.32 |  |  |  |  |  |  |
|  | | *tomkonis* | 1 | 3.08 | 3.08 | 0.51 | 0.51 | 0.32 | | 0.32 |  |  |  |  |  |  |
|  | | *salinitolerans* | 1 | 3.08 | 3.08 | 0.51 | 0.51 | 0.32 | | 0.32 |  |  |  |  |  |  |
| *Pantoea* | | *vagans* | 1 | 5.11 | 5.11 | 0.54 | 0.54 | 0.44 | | 0.44 |  |  |  |  |  |  |
|  | | *agglomerans* | 3 | 3.10 | 3.14 | 0.55 | 0.74 | 0.44 | | 0.64 |  |  |  |  |  |  |
|  | | *septica* | 1 | 3.10 | 3.10 | 0.56 | 0.56 | 0.42 | | 0.42 |  |  |  |  |  |  |
|  | | *wallisii* | 1 | 3.07 | 3.07 | 0.32 | 0.32 | 0.38 | | 0.38 |  |  |  |  |  |  |
| *Trinickia* | | *symbiotica ** | 3 | 3.16 | 3.16 | 0.78 | 0.79 | 0.71 | | 0.71 | 0.70 | 0.71 | 0.96 | 0.97 | 0.83 | 0.84 |
| *Dickeya* | | *chrysanthemi ** | 6 | 3.15 | 3.15 | 0.76 | 0.76 | 0.64 | | 0.65 | 0.40 | 0.40 | 0.73 | 0.73 |  |  |
|  | | *dianthicola ** | 72 | 3.14 | 3.15 | 0.74 | 0.75 | 0.64 | | 0.67 | 0.39 | 0.40 | 0.74 | 0.74 |  |  |
|  | | *dadantii ** | 3 | 3.15 | 3.15 | 0.76 | 0.76 | 0.64 | | 0.65 | 0.41 | 0.41 | 0.73 | 0.73 |  |  |
| *Pseudomonas* | | *floridensis* | 1 | 3.13 | 3.13 | 0.69 | 0.69 | 0.61 | | 0.61 |  |  |  |  |  |  |
|  | | *syringae* | 174 | 3.12 | 3.13 | 0.60 | 0.69 | 0.41 | | 0.56 |  |  |  |  |  |  |
|  | | *savastanoi* | 52 | 3.09 | 3.13 | 0.38 | 0.69 | 0.40 | | 0.56 |  |  |  |  |  |  |
|  | | *amygdali* | 7 | 3.13 | 3.13 | 0.68 | 0.69 | 0.54 | | 0.54 |  |  |  |  |  |  |
|  | | *cannabina* | 3 | 3.13 | 3.13 | 0.69 | 0.69 | 0.56 | | 0.56 |  |  |  |  |  |  |
|  | | *kribbensis* | 2 | 3.10 | 3.10 | 0.55 | 0.56 | 0.46 | | 0.47 |  |  |  |  |  |  |
|  | | *chlororaphis* | 93 | 3.10 | 3.10 | 0.56 | 0.56 | 0.45 | | 0.46 |  |  |  |  |  |  |
|  | | *bananamidigenes* | 1 | 3.10 | 3.10 | 0.56 | 0.56 | 0.46 | | 0.46 |  |  |  |  |  |  |
|  | | *glycinae* | 4 | 3.10 | 3.10 | 0.55 | 0.56 | 0.46 | | 0.46 |  |  |  |  |  |  |
|  | | *botevensis* | 1 | 3.10 | 3.10 | 0.55 | 0.55 | 0.46 | | 0.46 |  |  |  |  |  |  |
|  | | *fluorescens* | 8 | 3.10 | 3.10 | 0.55 | 0.56 | 0.44 | | 0.46 |  |  |  |  |  |  |
|  | | *crudilactis* | 1 | 3.10 | 3.10 | 0.55 | 0.55 | 0.45 | | 0.45 |  |  |  |  |  |  |
|  | | *brassicacearum* | 1 | 3.10 | 3.10 | 0.55 | 0.55 | 0.45 | | 0.45 |  |  |  |  |  |  |
|  | | *frederiksbergensis* | 1 | 3.10 | 3.10 | 0.55 | 0.55 | 0.45 | | 0.45 |  |  |  |  |  |  |
|  | | *granadensis* | 6 | 3.10 | 3.10 | 0.55 | 0.55 | 0.45 | | 0.46 |  |  |  |  |  |  |
|  | | *monsensis* | 1 | 3.10 | 3.10 | 0.55 | 0.55 | 0.44 | | 0.44 |  |  |  |  |  |  |
|  | | *ekonensis* | 1 | 3.10 | 3.10 | 0.56 | 0.56 | 0.45 | | 0.45 |  |  |  |  |  |  |
|  | | *asplenii* | 1 | 3.10 | 3.10 | 0.55 | 0.55 | 0.44 | | 0.44 |  |  |  |  |  |  |
|  | | *jessenii* | 1 | 3.10 | 3.10 | 0.55 | 0.55 | 0.45 | | 0.45 |  |  |  |  |  |  |
|  | | *tensinigenes* | 1 | 3.10 | 3.10 | 0.55 | 0.55 | 0.44 | | 0.44 |  |  |  |  |  |  |
| *Rhizobium* | | *tumorigenes* | 1 | 3.13 | 3.13 | 0.67 | 0.67 | 0.59 | | 0.59 |  |  |  |  |  |  |
|  | | *skierniewicense* | 2 | 3.08 | 3.08 | 0.50 | 0.50 | 0.33 | | 0.33 |  |  |  |  |  |  |
|  | | *lusitanum* | 1 | 3.08 | 3.08 | 0.49 | 0.49 | 0.35 | | 0.35 |  |  |  |  |  |  |
| *Xanthomonas* | | *arboricola* | 2 | 3.12 | 3.12 | 0.64 | 0.65 | 0.55 | | 0.55 |  |  |  |  |  |  |
| *Pectobacterium* | | *odoriferum* | 15 | 3.12 | 3.12 | 0.64 | 0.64 | 0.54 | | 0.54 |  |  |  |  |  |  |
|  | | *betavasculorum* | 2 | 3.12 | 3.12 | 0.64 | 0.64 | 0.54 | | 0.55 |  |  |  |  |  |  |
|  | | *actinidiae* | 4 | 3.12 | 3.12 | 0.64 | 0.64 | 0.54 | | 0.54 |  |  |  |  |  |  |
| *Acinetobacter* | | *pollinis* | 2 | 3.10 | 3.10 | 0.53 | 0.53 | 0.36 | | 0.36 |  |  |  |  |  |  |
| *Photorhabdus* | | *caribbeanensis* | 1 | 3.09 | 3.09 | 0.55 | 0.55 | 0.42 | | 0.42 |  |  |  |  |  |  |
|  | | *noenieputensis* | 1 | 3.09 | 3.09 | 0.55 | 0.55 | 0.42 | | 0.42 |  |  |  |  |  |  |
| *Xenorhabdus* | | *szentirmaii* | 2 | 3.08 | 3.09 | 0.39 | 0.53 | 0.42 | | 0.42 |  |  |  |  |  |  |
| *Liberibacter* | | *crescens* | 2 | 3.08 | 3.08 | 0.45 | 0.45 | 0.40 | | 0.40 |  |  |  |  |  |  |
| *Burkholderia* | | *vietnamiensis ** | 121 | 3.08 | 3.08 | 0.49 | 0.53 | 0.35 | | 0.36 | 0.69 | 0.70 | 0.92 | 0.93 |  |  |
|  | | *pyrrocinia* | 9 | 3.08 | 3.08 | 0.52 | 0.53 | 0.35 | | 0.36 |  |  |  |  |  |  |
|  | | *territorii* | 3 | 3.08 | 3.08 | 0.52 | 0.53 | 0.33 | | 0.35 |  |  |  |  |  |  |
|  | | *ubonensis ** | 1 | 3.08 | 3.08 | 0.53 | 0.53 | 0.36 | | 0.36 | 0.69 | 0.69 | 0.93 | 0.93 |  |  |
|  | | *cepacia* | 1 | 3.08 | 3.08 | 0.53 | 0.53 | 0.36 | | 0.36 |  |  |  |  |  |  |
|  | | *diffusa* | 6 | 3.08 | 3.08 | 0.53 | 0.53 | 0.32 | | 0.36 |  |  |  |  |  |  |
|  | | *seminalis* | 16 | 3.08 | 3.08 | 0.52 | 0.52 | 0.31 | | 0.36 |  |  |  |  |  |  |
|  | | *paludis* | 3 | 3.08 | 3.08 | 0.52 | 0.53 | 0.35 | | 0.35 |  |  |  |  |  |  |
|  | | *stabilis* | 7 | 3.08 | 3.08 | 0.52 | 0.52 | 0.33 | | 0.33 |  |  |  |  |  |  |
| *Noviherbaspirillum* | | *cavernae* | 1 | 3.05 | 3.05 | 0.36 | 0.36 | 0.34 | | 0.34 |  |  |  |  |  |  |
| *Streptomyces* | | *violaceoruber* | 1 | 3.04 | 3.04 | 0.30 | 0.30 | 0.34 | | 0.34 |  |  |  |  |  |  |
|  | | *californicus* | 5 | 3.04 | 3.04 | 0.30 | 0.30 | 0.33 | | 0.34 |  |  |  |  |  |  |
|  | | *mexicanus* | 1 | 3.04 | 3.04 | 0.30 | 0.30 | 0.31 | | 0.31 |  |  |  |  |  |  |
| *Rhodococcus* | | *jostii* | 1 | 3.04 | 3.04 | 0.30 | 0.30 | 0.33 | | 0.33 |  |  |  |  |  |  |
